# *miR-4484* suppresses hepatocellular carcinoma progression via targeting *KIF2C*

**DOI:** 10.1080/15476286.2025.2569192

**Published:** 2025-10-02

**Authors:** Jianyang Lin, Yun Cai, Zhihong Chen, Jichun Ma, Kun Zhao

**Affiliations:** a Department of General Surgery, Zhenjiang First People’s Hospital, Affiliated People’s Hospital of Jiangsu University, Zhenjiang, Jiangsu, China; b Center for Pharmacology and Toxicology, Institute for Pharmaco- and Toxicogenomic Research, Hanover Medical School (MHH), Hanover, Germany; c Department of Pharmacy, Key Laboratory for Major Obstetric Diseases of Guangdong Province, The Third Affiliated Hospital of Guangzhou Medical University, Guangzhou, Guangdong, China; d Department of Central Laboratory, Zhenjiang First People’s Hospital, Affiliated People’s Hospital of Jiangsu University, Zhenjiang, Jiangsu, China; e Department of Neurology, Zhenjiang First People’s Hospital, Affiliated People’s Hospital of Jiangsu University, Zhenjiang, Jiangsu, China; f Department of Cell Biology, Institute of Bioengineering, School of Medicine, Soochow University, Suzhou, Jiangsu, China

**Keywords:** antineoplastic, hepatocellular carcinoma, *KIF2C*, *miR-4484*, biomarker

## Abstract

Aberrantly expressed microRNA-4484 (*miR-4484*) has recently garnered attention for its involvement in human diseases, but its specific role in hepatocellular carcinoma (HCC) remains largely unexplored. This study investigates the function of *miR-4484* in HCC progression and its regulatory interaction with *KIF2C*. Analysis of the data from the TCGA-LIHC database revealed that *miR-4484* expression is significantly downregulated in HCC tissues, with lower levels correlating with worse prognosis. *In vitro* experiments confirmed that *miR-4484* expression is lower in HCC cell lines compared to a normal liver cell line. Functional assays demonstrated that *miR-4484* overexpression via a *miR-4484* mimic suppressed cell proliferation and induced G1 phase arrest, whereas *miR-4484* inhibition promoted proliferation and facilitated cell cycle progression from G1 to S and G2 phases. Additionally, *KIF2C* expression was significantly upregulated in HCC tissues and cell lines, exhibiting an inverse correlation with *miR-4484* levels. Dual-Luciferase Reporter Assays confirmed that *miR-4484* directly binds to *KIF2C*, thereby regulating its expression and influencing cell proliferation and cell cycle progression. *In vivo*, subcutaneous intratumoral injection of the *miR-4484* mimic in nude mice significantly inhibited HCC tumour growth. These findings highlight *miR-4484* as a potential tumour suppressor in HCC through its direct targeting of *KIF2C*, underscoring its promise as a therapeutic target for HCC treatment.

## Introduction

The global mortality rate of liver cancer rose from the third highest in 2018 to the second highest in 2020 of all cancers [1,2]. As the most common histologic type of primary liver tumour, HBV infection and alcohol consumption account for over 80% of hepatocellular carcinoma (HCC) deaths [3]. Surgery to remove the tumour, which has a five-year survival rate of 60–70%, and transplantation, which has a four-year survival rate of 70–80%, are the preferred treatments for HCC patients. Transcatheter arterial chemoembolization or radiotherapy, rarely results in cures but provides survival benefits [4]. Besides the traditional chemotherapeutic agents, targeted therapy such as anti-angiogenesis inhibitors or anti-PD-1/PD-L1, have made significant advancements. However, the overall outcomes have not yet been fully reported [5]. HCC is highly heterogeneous and refractory to several standard methods of treatment, leading to a high recurrence rate and poor prognosis [6]. Effective diagnostic biomarkers and antineoplastic targets must be identified [7].

*MicroRNAs* (miRNAs) are small, conserved, non-coding RNAs ( < 25 nucleotides) that regulate gene expression post-transcriptionally, often through interactions with the 3′-UTR of target mRNAs [8]. These molecules are essential for diverse biological processes and are closely linked to tumorigenesis and cancer progression. Xu Y et al. reported that human umbilical cord mesenchymal stem cells-derived exosomal *microRNA-451a* repress the epithelial-mesenchymal transition of HCC by inhibiting ADAM10 [9]. A systematic investigation based on microRNA-mediated gene regulatory networks reveals that dysregulation of the *microRNA-19a*/Cyclin D1 axis confers oncogenic potential and worse prognosis in human HCC [10]. Detecting and modulating miRNAs offers significant diagnostic and therapeutic potential [11–13].

*miR-4484*, a tumour suppressor, is often silenced or downregulated in cancers including Glioblastoma Multiforme [14], Lymphoma [15], Breast cancer [16], and Oral squamous cell carcinoma [17]. It targets genes like *ABCA13*, *S100A2*, *DAG1*, *CDK4*, *STC1*, *E2F2*, *ITGB1*, *PXDN*, and *ZBTB20*, affecting cancer cell migration, invasion, cell cycle progression, and metastasis [14]. Zhong HY et al. identified the regulatory role of *miR-4484* on integrin α6 in gastric cancer [18], while Devis Pascut et al. reported reduced serum *miR-4484* levels in HCC, distinguishing HCC-positive cases from HCC-negative ones [19]. These results suggest that *miR-4484* is a potential biomarker for HCC detection, but its molecular mechanisms require further exploration.

*KIF2C* (kinesin family member 2C), also known as *MCAK* (mitotic centromere-associated kinesin), plays a crucial role in the progression of various cancers, particularly HCC [20,21]. As a microtubule depolymerase, *KIF2C* is essential for proper chromosomal segregation during mitosis. It regulates microtubule attachment at kinetochores and facilitates chromosome alignment and separation, maintaining genomic stability [22]. When *KIF2C* is dysregulated, it disrupts its functions and causes genomic instability and aneuploidy, which are key drivers of tumorigenesis. *KIF2C* is frequently upregulated in various cancers, including HCC, where its high expression is associated with increased cell proliferation, invasion, and migration. This contributes to disease progression and metastasis [21,23]. Moreover, *KIF2C* plays a pivotal role in cancer development by modulating key signalling pathways, such as PI3K/AKT/MAPK [24], Wnt/β-catenin [25], mTORC1 [25], and Ras/MAPK [26], all of which regulate cell survival, proliferation, and migration. Its overexpression enhances the activation of these pathways, driving a more aggressive and malignant phenotype in HCC cells.

For these reasons, *KIF2C* represents a promising target for therapeutic intervention, as its inhibition could restore normal cell cycle progression and reduce tumour growth. Investigating how *KIF2C* is regulated, particularly by miRNAs like *miR-4484*, may offer valuable insights into potential therapeutic strategies for HCC treatment.

## Materials and methods

### Ethics statement

This study was conducted in accordance with the principles of the Declaration of Helsinki. The detection of samples and procedures for cell and animal experiments were approved by the Ethics Committee of Jiangsu University Affiliated People’s Hospital. All animal experiments were performed using nude mice, following the guidelines outlined in the Guide for the Care and Use of Laboratory Animals by the National Institutes of Health.

### Bioinformatics analysis

The expression data for *miR-4484* and *KIF2C* in HCC and normal liver tissues were obtained from the Liver Hepatocellular Carcinoma (LIHC) project of The Cancer Genome Atlas (TCGA) database. R software (Version 4.3.2) was used for data processing and statistical analysis. The R packages survival (Version 3.8–3) and survminer (Version 0.5.0) were utilized to perform Kaplan-Meier survival analysis, assessing the association between *miR-4484* expression and overall survival (OS) of HCC patients. Additionally, the ggpubr package (Version 0.6.1) was employed for visualizing differences in *miR-4484* expression across various clinicopathological subtypes, while statistical comparisons were conducted using the rstatix package (Version 0.7.2).

To predict potential target genes of *miR-4484*, three publicly available miRNA target prediction databases were used: TargetScan (https://www.targetscan.org/vert_80/), miRTarBase (https://awi.cuhk.edu.cn/~miRTarBase/miRTarBase_2025/php/index.php), and miRWalk (http://mirwalk.umm.uni-heidelberg.de/search_mirnas/). The overlapping target genes identified across these databases were visualized using a Venn plot, generated with the R package VennDiagram (Version 1.7.3). *KIF2C* expression between tumour and normal tissues was compared using box plots. Scatter plots with fitted regression lines were generated to visualize the association between *KIF2C* and *miR-4484*.

### Cell culture

This study evaluated four HCC cell lines: HepG2, Hep3B, Huh7, and SMMC-7721, as well as one normal liver cell line, LO2. All cell lines were obtained from the American Type Culture Collection (ATCC, Rockville, MD, USA). The cells were cultured in Dulbecco’s Modified Eagle Medium (DMEM) with 10% foetal bovine serum (FBS) (Cat#16000–044, Gibco), along with 100 U/mL penicillin and 100 μg/mL streptomycin to prevent bacterial contamination and maintain a sterile environment. The cells were maintained at 37°C in a humidified atmosphere containing 5% CO₂. The medium was refreshed every 2 ~ 3 days, and cells were subcultured when they reached approximately 80 ~ 90% confluence. This standardized culture condition ensured optimal cell growth and consistency across experiments.

### Cell transfection

Human-specific *miR-4484* mimic, inhibitor, and their relative negative controls (miR-NC), were obtained from GenePharma (Shanghai, China). The RNA sequence of *miR-4484*, *miR-4484* mimic, *miR-4484* inhibitor was shown in Table S1. The mimic and inhibitor were transfected into Huh7 and Hep3B cells, respectively, at a confluence of 60 ~ 70% using Lipo2000 (Cat#11668019, Invitrogen, Carlsbad, CA, USA). A total of 5 µL (100 pmol) of the *miR-4484* mimic, *miR-4484* inhibitor, or miR-NC was diluted in 245 µL of serum-free Optimized Minimum Essential Medium (Opti-MEM; Cat# 51,985–034, Gibco). And then, 15 µL of Lipo2000 was diluted in 735 µL of Opti-MEM, mixed well, and incubated at room temperature for 5 minutes. Then, 250 µL of the Lipo2000 mixture was added to the Opti-MEM containing the *miR-4484* mimic or *miR-4484* inhibitor or miR-NC, gently mixed, and incubated for an additional 20 minutes at room temperature to form complexes. Before adding the transfection complexes, cells were washed once with sterile PBS, and the culture medium was replaced with 2 mL of serum-free DMEM. Next, the transfection complexes were carefully added to the culture wells, and the cells were incubated at 37°C with 5% CO₂ for 6 hours. After this period, the transfection medium was removed and replaced with complete DMEM containing 10% FBS. Following 48 hours of transfection, cells were harvested for quantitative Real-Time-PCR (qRT-PCR) and Western Blot (WB) analysis to verify the transfection efficiency and evaluate the expression of *miR-4484* and *KIF2C*.

### qRT-PCR

Total RNA was extracted using TRIzol Reagent (Cat#1596–026, Invitrogen) and reverse-transcribed into cDNA with the RevertAid First Strand cDNA Synthesis Kit (Cat#K1622, Fermentas, Thermo Fisher Scientific Inc., Carlsbad, CA, USA). For qRT-PCR, 1 ug of RNA was used per reaction to ensure consistent and accurate measurements. mRNA expression was detected using SYBR Master Mix (Cat#K0223, Thermo Fisher Scientific Inc.), and the primers are listed in Table S2. qRT-PCR was performed on an ABI 7300 Fast Real-Time PCR System (Applied Biosystems, Carlsbad, CA, USA). Relative expression levels of *miR-4484* and *KIF2C* were calculated using the 2^−ΔΔCt method, with results normalized to U6 and GAPDH, respectively.

### Western blot

The protein content of cell or tissue samples was quantified using the Bicinchoninic Acid (BCA) Protein Assay Kit (Cat#PICPI23223, Thermo Fisher Scientific Inc., USA) after lysis with radioimmunoprecipitation assay (RIPA) buffer (Cat#BYL40825, JRDUN Biotech, Shanghai, China). Each sample contained 20 μg of protein, which was separated by 10% sodium dodecyl sulphate-polyacrylamide gel electrophoresis (SDS-PAGE). The proteins were transferred to an activated polyvinylidene fluoride (PVDF) membrane. They were then incubated with primary antibodies at 4°C for up to 12 hours. The antibodies used were anti-KIF2C (Cat#28372–1-AP, 1:1000) and GAPDH (Cat#60004–1-1 G, 1:5000) from Proteintech Group Inc., Rosemont, IL, USA. Following membrane washing with PBS, goat anti-rabbit IgG H&L secondary antibody (Cat# A0208, Beyotime Biotechnology, Jiangsu, China) was applied at a 1:1000 dilution for KIF2C, and goat anti-mouse IgG H&L secondary antibody (Cat# A0216, Beyotime Biotechnology, Jiangsu, China) was applied at a 1:1000 dilution for GAPDH. Blots were developed using an Enhanced Chemiluminescence Kit (Millipore, Milford, MA, USA), and image quantification was performed using ImageJ software (version 1.47 v, National Institutes of Health, USA). The expression levels of KIF2C were normalized to GAPDH.

### Cell proliferation assay

Relative cell viability was assessed using the Cell Counting Kit-8 (CCK-8) assay (Cat# CP002, SABiosciences, Qiagen, CA, USA). Cells were seeded in triplicate wells for each group at a density of 3 × 10^3^ cells per well in 96-well plates. They were then cultured overnight at 37°C in a humidified incubator with 5% CO₂. At 0 h, 12 h, 24 h, 48 h, and 72 h post-transfection, CCK-8 reagent was mixed with serum-free essential medium at a 1:10 ratio. Then, 100 µL of this mixture was added to each well. The plates were incubated for 1 h at 37°C in the same incubator. Absorbance at 450 nm was measured using a microplate reader (Cat# DNM-9602, Pulangxin, Beijing, China), and the corresponding values for each group were recorded. Cell proliferation was subsequently quantified based on the 450 nm absorbance readings.

### Cell cycle analysis

Cells were harvested 48 hours after transfection and fixed with 70% ethanol at 4°C, following the protocol provided by the Cell Cycle and Apoptosis Analysis Kit (Cat#C1052, Beyotime Biotechnology, Jiangsu, China). Cell cycle distribution was analysed using a CytoFLEX flow cytometer (Beckman Coulter Inc., Brea, CA, USA), which detected red luminescence at 488 nm through the FL2 channel. The data were analysed using FlowJo software and presented as percentages. Freq.G1, Freq.S, and Freq.G2 represent the proportions of cells in the G1, S, and G2 phases, respectively.

### Luciferase reporter assay

The *KIF2C* sequence (NM_001297655) was obtained from the National Center for Biotechnology Information (NCBI), and its 3’-UTR region was selected for target analysis. The pGL3-Promoter vector, containing the firefly luciferase gene (luc2), and the pRL-TK vector, containing the Renilla luciferase gene (hRluc), were used for this assay. The 3’-UTR of *KIF2C* was amplified and cloned into the pGL3-Promoter vector to create the pGL3-Promoter-wt*KIF2C* plasmid. To confirm the interaction between hsa-*miR-4484* and *KIF2C*, site-directed mutagenesis was used to disrupt the *miR-4484* binding site, generating the pGL3-Promoter-mut*KIF2C* plasmid. The sequences of wt*KIF2C* 3‘-UTR and mut*KIF2C* 3’−UTR were provided in Table S3.

Huh7 cells were seeded in 6-well plates at 5 × 10^5^ cells per well and incubated at 37°C for 24 hours. After cell adherence, transfection was performed using Lipo2000. Each group received 1.5 µg of firefly luciferase plasmid and 20 ng of Renilla luciferase plasmid. In the NC group, 5 µL of NC-miRNA (100 pmol) was added to the wild-type luciferase plasmid medium. In the Mimic+wt group, 5 µL of hsa-*miR-4484* mimic (100 pmol) was added to the wild-type plasmid medium. For the Mimic+mut group, 5 µL of hsa-*miR-4484* mimic (100 pmol) was added to the mutant plasmid medium, with each group performed in triplicate. 15 µL of Lipo2000 was diluted in 735 µL of Opti-MEM and incubated at room temperature for 5 minutes. Each RNA/DNA mixture was combined with 250 µL of the Lipo2000 mixture, incubated for 20 minutes, and then added to the cells after replacing the medium with 1 mL of serum-free medium. After incubating at 37°C for 6 hours, the transfection medium was replaced with complete DMEM containing 10% FBS, and the cells were cultured for an additional 48 hours. After 48 hours, cells were lysed with 500 µL of Passive Lysis Buffer (PLB) and gently shaken at room temperature for 15 minutes. Luciferase activity was measured using the Dual-Luciferase Reporter Assay System (Cat#E1910, Promega, Fitchburg, WI, USA). The ratio of firefly luciferase activity to Renilla luciferase activity was calculated for further analysis.

### KIF2C overexpression plasmid construction and cell transfection

To investigate the function of *KIF2C*, we constructed a eukaryotic overexpression vector. The pCDNA3.1(+) vector (Cat# 89,496, Addgene, Watertown, MA, USA) and competent DH5α cells (TransGen Biotech Co., Ltd) were utilized. A diagram of the pCDNA3.1(+) vector structure is provided in Table S4. We synthesized the coding sequence (CDS) region of the *KIF2C* gene (NM_006845.4), which spans 2178 bp, incorporating restriction sites upstream and downstream based on the vector’s restriction site information (Hind III and EcoR I). The sequence of the *KIF2C* containing the restriction sites was synthesized by Genewiz Gene Company. The primer sequences were designed as follows, with the restriction sites underlined:

KIF2C-F: 5’-CCCAAGCTTATGGCCATGGACTCGTCG-3’ (Hind III)

KIF2C-R: 5’-CGGAATTCTCACTGGGGCCGTTTCTTG-3’ (EcoR I)

The synthesized plasmid was sent to Shanghai Meiji Biotech for DNA sequencing. The sequencing results were aligned with the *KIF2C* reference sequence in NCBI. A plasmid that matched 100% was kept in bacterial solution. Plasmid extraction was performed using the Beijing Solebow Plasmid Extraction Kit. Sequencing confirmed there were no mismatches, deletions, or reverse connections in the *KIF2C* sequence, confirming the successful construction of the *KIF2C* recombinant eukaryotic overexpression vector (oeKIF2C).

To validate the effect of the oeKIF2C, we transfected Huh7 cells with oeKIF2C or blank vector. The experiment included three groups: Control group: 250 µL of Opti-MEM. Vector group: 5 ul (100 pmol) of blank vector was dissolved in 245 µL of Opti-MEM. oeKIF2C group: 5 ul (100 pmol) of oeKIF2C was dissolved in 245 µL of Opti-MEM. Following cell transfection was performed according to the standard transfection protocol (present in the cell transfection section).

### In vivo experiments

Twelve female BALB/c athymic nude mice, aged four to five weeks (weighing 15–17 g), were obtained from Shanghai Slack Laboratory Animals Co., Ltd. After one week of pathogen-free acclimatization, each mouse received a subcutaneous injection of 5 × 10^6^ Huh7 cells. These cells were in the exponential growth phase and suspended in 100 μL of PBS. Twelve days post-injection, the tumour-bearing mice were randomly divided into two groups. One group received 0.5 oligonucleotide dose (OD) of *miR-4484* mimic, and the other received miR-NC, injected directly into the tumours every day for three weeks. Tumour volumes were measured before each injection with a Vernier caliper, using the formula: 0.5 × length × width^2^ for calculation. After 21 days of intratumoral injection, all animals were sacrificed on Day 33, and solid tumours were collected for further analysis. Tumour weights were recorded, and samples underwent haematoxylin-eosin staining (HE staining). Expression levels of *miR-4484* and KIF2C were assessed by qRT-PCR and WB. All efforts were made to minimize animal suffering throughout the whole experiment.

### Statistical analysis

Continuous variables are expressed as Mean ± Standard Deviation (SD). Data following a normal distribution were analysed using two-tailed unpaired t-tests. For non-normally distributed data, the Mann-Whitney test was employed. Differences among three or more groups were assessed using one-way analysis of variance (ANOVA), followed by Tukey’s multiple comparisons test. If the assumption of variance homogeneity was not met, the Kruskal-Wallis test was applied, accompanied by Dunn’s multiple comparisons test. The correlation between *KIF2C* expression and *miR-4484* was assessed by Spearman’s correlation analysis. *p <* 0.05 was considered statistically significant.

## Results

### miR-4484 expression in HCC: clinical relevance, quantification, and functional validation

**Figure 1.**
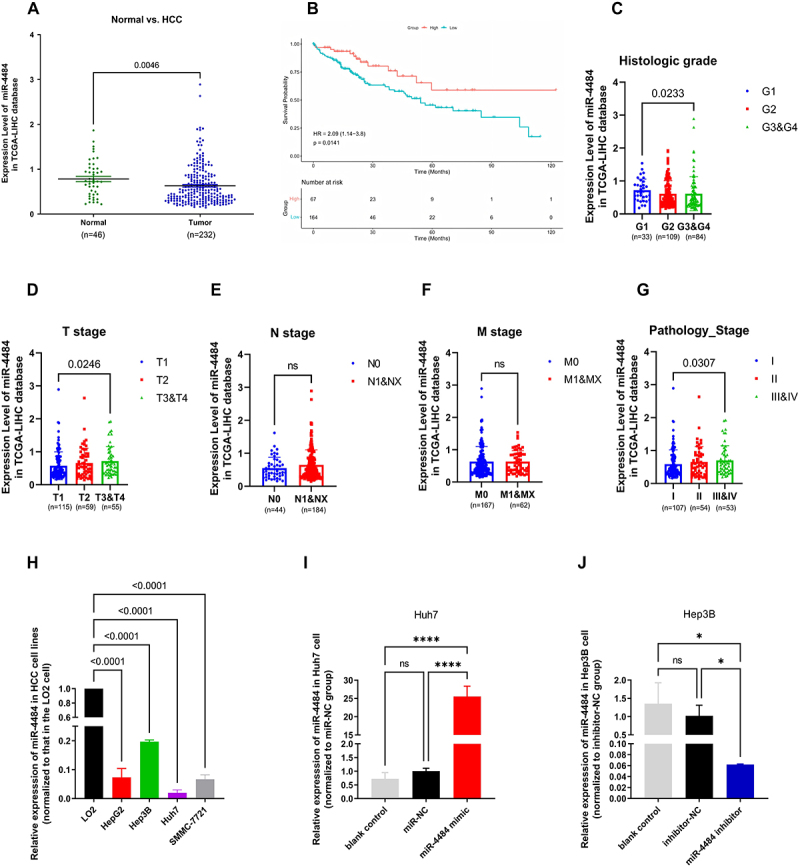
*miR-4484* expression in HCC: clinical relevance, quantification, and functional validation. (A) Analysis of the TCGA-LIHC database showed that *miR-4484* expression was significantly lower in HCC tissues than in normal tissues (*p =* 0.0046). (B) Kaplan-Meier survival analysis indicated that patients with low *miR-4484* expression had significantly worse survival outcomes than those with high *miR-4484* expression (HR = 2.09, *p =* 0.0141). (C) *miR-4484* expression was compared across different histologic grades: G1 (well-differentiated), G2 (moderately differentiated), and G3&G4 (poorly differentiated and undifferentiated). A significant reduction in expression was observed as the histological grade increased from G1 to G3&G4 (*p =* 0.0233). (D)*miR-4484* expression was evaluated based on tumor size and invasion (T stage). The classifications are as follows: T1 (single tumor ≤5 cm, no vascular invasion), T2 (single tumor > 2 cm with vascular invasion or multiple tumors ≤5 cm), and T3&T4 (multiple tumors, at least one > 5 cm or significant invasion). A significant elevation in expression was observed from T1 to T3&T4 (*p =* 0.0246).0 (E) *miR-4484* expression was evaluated based on lymph node metastasis (N stage). No significant differences were noted in *miR-4484* expression concerning the N stage. (F) *miR-4484* expression was evaluated based on distant metastasis stage (M stage). No significant differences were noted in *miR-4484* expression concerning the M stage. (G) *miR-4484* expression levels were analyzed across different pathological stages: stage I, stage II, and stages III&IV (advanced stages). A significant increase in expression was observed from stage I to stages III&IV (*p =* 0.0307). (H) *miR-4484* expression was quantified in HCC cell lines (HepG2, Hep3B, Huh7, SMMC-7721) and normal liver cells (LO2) by qRT-PCR. U6 was used as the normalization control. All experiments were performed in triplicate. The results indicated that *miR-4484* expression was significantly downregulated in all HCC cell lines relative to LO2 (*p <* 0.0001 for all comparisons). Among the HCC cell lines, Huh7 exhibited the lowest expression, followed by SMMC-7721, HepG2, and Hep3B. (I) *miR-4484* expression was measured in Huh7 cells transfected with a *miR-4484* mimic (100 pmol) or miR-NC (100 pmol) by qRT-PCR. Huh7 was used as the blank control. *n* = 3 per group. U6 was used as the normalization control. The results showed significantly increased *miR-4484* expression in the *miR-4484* mimic group compared to the miR-NC group. ****p < 0.0001. (J) *miR-4484* expression was quantified in Hep3B cells transfected with a *miR-4484* inhibitor (100 pmol) or miR-NC (100 pmol) by qRT-PCR. Hep3B was used as the blank control. *n* = 3 per group. U6 was used as the normalization control. Results showed a significant decrease in *miR-4484* expression in the *miR-4484* inhibitor group compared to the inhibitor-NC group. *p < 0.05.

We analysed the expression profiles of *miR-4484* using the TCGA-LIHC database. Samples with missing values (NA) were excluded from this analysis. The analysis included 46 normal tissue samples and 232 HCC tumour tissue samples (Table S5). The results indicated that *miR-4484* expression levels were significantly lower in HCC tissues than in normal tissues (Figure 1(A)). After excluding samples with missing values, 231 tumour cases with both *miR-4484* expression data and OS information were included for survival analysis (Table S6). Using the surv cutpoint function in R, we determined an optimal cut-off value of 0.7628 for *miR-4484* expression. Kaplan-Meier survival analysis showed that patients with low *miR-4484* expression had significantly worse survival outcomes than those with high *miR-4484* expression (Figure 1(B)). Next, we compared *miR-4484* expression levels across different pathological subgroups (Table S7). Compared to the G1 stage, *miR-4484* expression was significantly lower in the G2 and G3&G4 groups (Figure 1(C)), suggesting that poorer differentiation of liver cancer tissues was associated with decreased *miR-4484* expression. In contrast, an increase in *miR-4484* expression was observed with advanced tumour staging (T3&T4) (Figure 1(D)). No significant differences were noted in *miR-4484* expression concerning the lymph node involvement stage (N stage) (Figure 1(E)) or distant metastasis stage (M stage) (Figure 1(F)). Moreover, we found that *miR-4484* expression was significantly higher in Stage III&IV than in Stage I (Figure 1(G)).

To further validate these findings, we quantified *miR-4484* expression in HCC cell lines (HepG2, Hep3B, Huh7, and SMMC-7721) and a normal liver cell line (LO2) using qRT-PCR. The results confirmed that *miR-4484* expression was significantly downregulated in all HCC cell lines compared to LO2, with p-values < 0.0001 for all comparisons (Figure 1(H)). Among the HCC cell lines, Huh7 showed the lowest *miR-4484* expression, followed by SMMC-7721, HepG2, and Hep3B.

To assess the functional regulation of *miR-4484*, we selected Huh7 cells (lowest *miR-4484* expression) for overexpression experiments and Hep3B cells (highest *miR-4484* expression) for inhibition experiments. The *miR-4484* mimic and inhibitor were transfected into Huh7 and Hep3B cells, respectively. In the *miR-4484* mimic group, *miR-4484* expression was significantly upregulated compared to the miR-NC group (*p <* 0.0001) (Figure 1(I)). Conversely, in the *miR-4484* inhibitor group, *miR-4484* expression was significantly downregulated compared to the inhibitor-NC group (Figure 1(J), *p <* 0.05). These results confirm that both the mimic and inhibitor effectively regulate *miR-4484* expression and are suitable for further research.

### Cellular effects of miR-4484 mimic or inhibition on HCC

**Figure 2.**
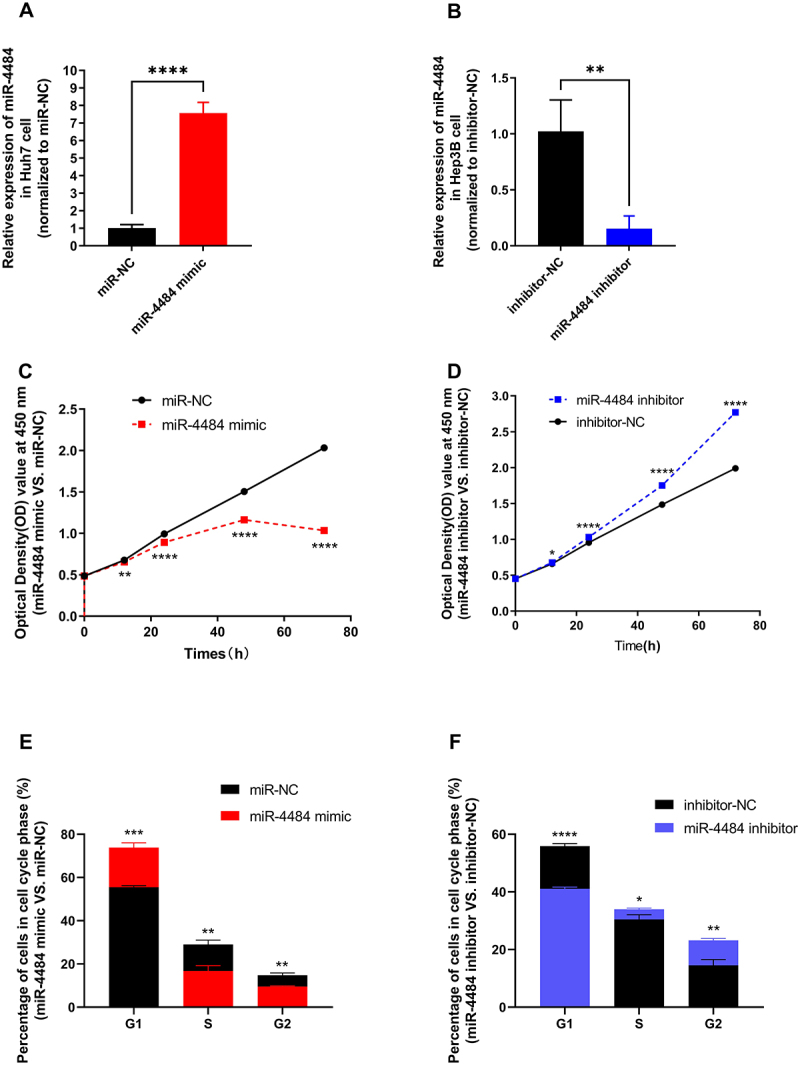
Cellular effects of *miR-4484* overexpression or inhibition on HCC. (A) Huh7 cells treated with a *miR-4484* mimic (100 pmol) showed a significant increase in *miR-4484* expression compared to the miR-NC group, as measured by qRT-PCR. *n* = 3 per group. U6 was used as the normalization control. (B) Hep3B cells transfected with a *miR-4484* inhibitor (100 pmol) showed a significant decrease in *miR-4484* expression compared to the inhibitor-NC group, as measured by qRT-PCR. *n* = 3 per group. U6 was used as the normalization control. (C) the CCK-8 assay was used to measure cell proliferation in Huh7 cells transfected with the *miR-4484* mimic. The *miR-4484* mimic group exhibited significantly reduced proliferation compared to the miR-NC group at 12 h, 24 h, 48 h, and 72 h. (D) Hep3B cells treated with the *miR-4484* inhibitor showed significantly enhanced cell proliferation compared to the inhibitor-NC group at 12 h, 24 h, 48 h, and 72 h. (E) Flow cytometry analysis showed that Huh7 cells treated with the *miR-4484* mimic had a significantly higher proportion of cells in the G1 phase, while the S and G2 phase populations significantly decreased. (F) Flow cytometry analysis of Hep3B cells transfected with the *miR-4484* inhibitor showed a marked reduction in the proportion of cells in the G1 phase, accompanied by significant increases in the S and G2 phase populations.

Cell transfection experiments were conducted to evaluate the effects of *miR-4484* on cell proliferation and cell cycle distribution in HCC cell lines. In Huh7 cells, transfection with a *miR-4484* mimic significantly upregulated *miR-4484* expression compared to the miR-NC group (Figure 2(A), *p <* 0.0001). Conversely, in Hep3B cells, transfection with a *miR-4484* inhibitor significantly downregulated *miR-4484* expression (Figure 2(B), *p <* 0.01). CCK-8 assays showed that *miR-4484* mimic treatment significantly decreased cell proliferation viability in Huh7 cells at 12 h, 24 h, 48 h, and 72 h (Figure 2(C), *p <* 0.01), whereas the *miR-4484* inhibitor significantly increased cell proliferation viability in Hep3B cells over the same time periods (Figure 2(D), *p <* 0.05). Flow cytometry analysis revealed that the *miR-4484* mimic induced G1 phase arrest in Huh7 cells by increasing the percent of cells in the G1 phase and decreasing those in the S and G2 phases, thereby inhibiting the G1 to S transition (Figure 2(E)). In contrast, the *miR-4484* inhibitor promoted cell cycle progression in Hep3B cells by reducing the percent of cells in the G1 phase and increasing those in the S and G2 phases, facilitating the transition from G1 to S to G2 (Figure 2(F)). Collectively, these findings highlight the critical role of *miR-4484* in regulating cell proliferation, and cell cycle progression in HCC cells.

*p < 0.05; **p < 0.01; ***p < 0.001; ****p < 0.0001.

### miR-4484 targets KIF2C and negatively regulates its expression in HCC

**Figure 3.**
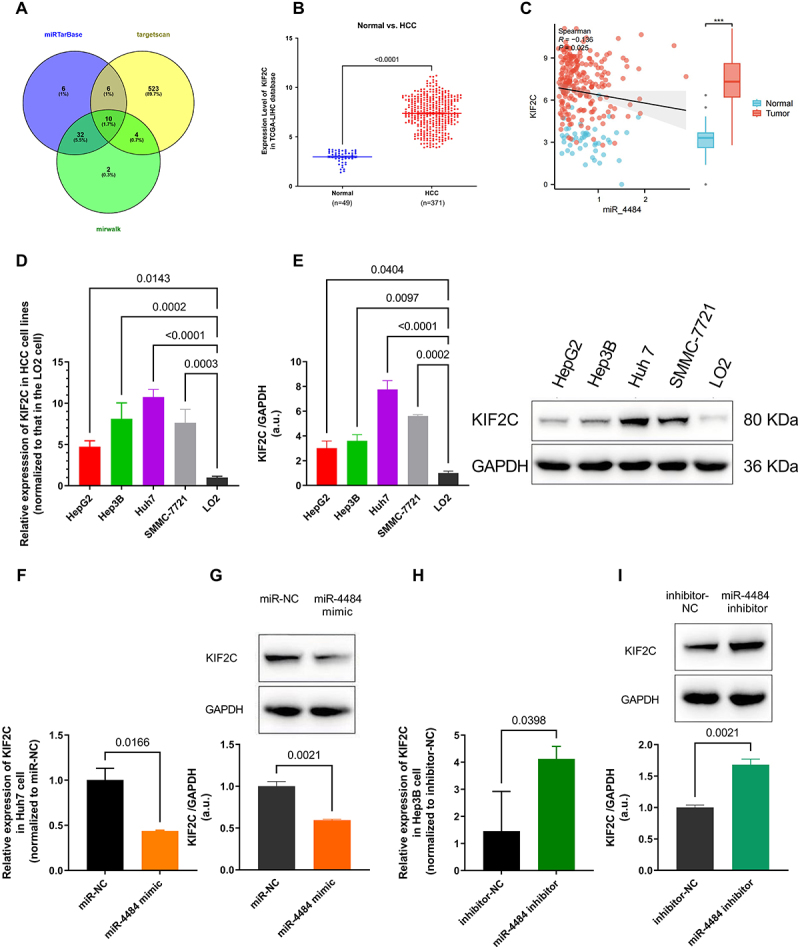
*miR-4484* targets *KIF2C* and negatively regulates its expression in HCC. (A) Venn plot identified 10 common genes shared across three miRNA target prediction databases: TargetScan, miRtarbase, and miRwalk. (B) Based on the analysis of TCGA-LIHC data, we demonstrated that *KIF2C* expression was significantly higher in HCC tissues compared to normal tissues (*p <* 0.0001). (c) Spearman correlation analysis demonstrated a significant negative correlation between *miR-4484* and *KIF2C* expression, suggesting a potential regulatory interaction between the two (*R* = −0.136, *p =* 0.025). (D) *KIF2C* expression was quantified in HCC cell lines and the normal liver cell line LO2 using qRT-PCR. Significantly elevated *KIF2C* expression was found in all HCC cell lines compared to LO2 (*p <* 0.05 for all comparisons). All experiments were performed in triplicate. Among the HCC cell lines, Huh7 showed the highest *KIF2C* expression, followed by Hep3B, SMMC-7721, and HepG2. (E) KIF2C protein expression in HCC cell lines and LO2 was analyzed by WB, using GAPDH as the internal control. All experiments were performed in triplicate. The KIF2C/GAPDH ratios were calculated. The results were normalized to the average value of LO2 and visualized. The left panel shows the quantification results, while the right panel displays representative WB images. KIF2C protein levels were significantly elevated in all HCC cell lines compared to LO2 (*p <* 0.05 for all comparisons), with Huh7 showing the highest protein expression, followed by Hep3B, SMMC-7721, and HepG2. (F) *KIF2C* expression in Huh7 cells transfected with a *miR-4484* mimic was analyzed using qRT-PCR. *n* = 3 per group. GAPDH was used as the normalization control. The results indicated a significant reduction in *KIF2C* expression in the *miR-4484* mimic group compared to the miR-NC group (*p =* 0.0166). (H) *KIF2C* expression in Hep3B cells treated with the *miR-4484* inhibitor was assessed using qRT-PCR. The *miR-4484* inhibitor group demonstrated significantly increased *KIF2C* expression compared to the inhibitor-NC group (*p =* 0.0398). (I) KIF2C protein expression in Hep3B cells transfected with the *miR-4484* inhibitor was analysed by WB, using GAPDH as the internal control. All experiments were performed in triplicate. Quantification showed that KIF2C protein levels were markedly increased in the *miR-4484* inhibitor group compared with the inhibitor-NC group (*p =* 0.0021).

To explore the molecular mechanism of *miR-4484* in HCC, we integrated data from three miRNA target prediction databases to identify its potential target genes. A total of 2,627 predicted target genes were retrieved from TargetScan, with 543 genes (cumulative weighted context++ score < −0.2) selected for further analysis (Table S8). Additionally, 48 experimentally validated target genes were obtained from miRWalk (Table S9), and 54 predicted target genes were identified in miRTarBase (Table S10). Venn diagram analysis revealed 10 common target genes across all three datasets, including *KIF2C*, *SERINC5*, *GABARAP*, *CYP1B1*, *PEBP1*, *PTCH2*, *LITAF*, *CERS2*, *ZNF791*, and *APP* (Table S11, Figure 3(A)). Among them, *KIF2C* was selected as the most relevant predicted target gene for further investigation.

Using the TCGA-LIHC database, we analysed *KIF2C* expression profiles, excluding samples with missing values. The dataset included 49 normal tissue samples and 371 HCC tumour samples (Table S12). The results showed that *KIF2C* expression was significantly higher in HCC tissues than in normal tissues (Figure 3(B), *p <* 0.0001), exhibiting an inverse expression pattern compared to *miR-4484*. To further validate the relationship between *miR-4484* and *KIF2C*, we performed Spearman correlation analysis using data from 227 HCC tumour tissue samples with available expression profiles for both *miR-4484* and *KIF2C* (Table S13). The results demonstrated a significant negative correlation between *miR-4484* and *KIF2C* expression (Figure 3(C), *p <* 0.05), suggesting a potential regulatory interaction between the two.

To validate these findings at the cellular level, we quantified KIF2C expression in HCC cell lines (HepG2, Hep3B, Huh7, SMMC-7721) and the normal liver cell line (LO2) using qRT-PCR (Figure 3(D)) and WB (Figure 3(E)). The results confirmed that KIF2C expression was significantly upregulated in all HCC cell lines compared to LO2 (*p <* 0.05). Among the HCC cell lines, Huh7 exhibited the highest KIF2C expression, followed by Hep3B, SMMC-7721, and HepG2.

To further investigate the regulatory relationship between *miR-4484* and *KIF2C*, we performed *miR-4484* overexpression and inhibition experiments. Transfection of the *miR-4484* mimic in Huh7 cells significantly downregulated *KIF2C* expression at both the mRNA (Figure 3(F), *p <* 0.05) and protein levels (Figure 3(G)). Conversely, *miR-4484* inhibition in Hep3B cells led to a significant upregulation of KIF2C expression at both the mRNA (Figure 3(H), *p <* 0.05) and protein levels (Figure 3(I)). These findings support the hypothesis that *miR-4484* negatively regulates KIF2C expression, potentially by directly targeting *KIF2C* mRNA for degradation, leading to reduced protein levels. However, further mechanistic studies are required to confirm this interaction and fully elucidate the regulatory role of *miR-4484* in HCC.

### KIF2C is confirmed as a target of miR-4484, highlighting its importance in the regulatory pathway

**Figure 4.**
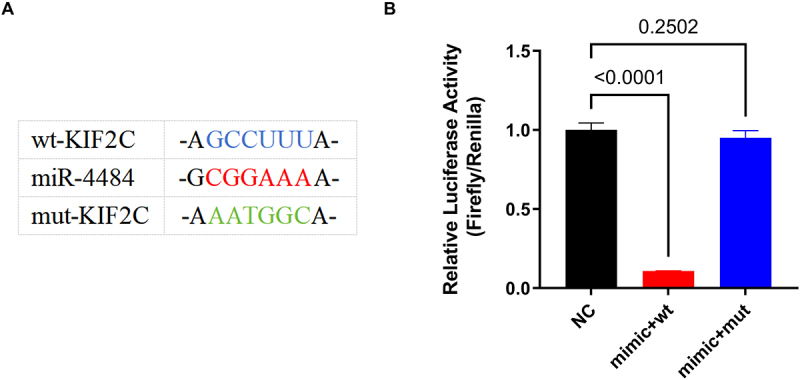
*miR-4484* inhibits HCC progression by targeting *KIF2C*. (A) Schematic illustration depicting the predicted binding site of *miR-4484* within the wild-type (wt) and mutated (mut) KIF2C 3′-UTR. The wild-type sequence shows a direct interaction between *miR-4484* and *KIF2C*, which is disrupted in the mutated version. (B) the dual-luciferase Reporter assay confirmed *miR-4484’*s direct interaction with *KIF2C*. Relative luciferase activity (Firefly/Renilla) was significantly reduced in the mimic+wt group compared to the NC group (*p <* 0.0001), indicating that *miR-4484* effectively binds to and represses *KIF2C* via its 3′-UTR. Conversely, luciferase activity in the mimic+mut group was comparable to the NC group (*p =* 0.2502).

We conducted a Dual-Luciferase Reporter Assay to examine the interaction between *miR-4484* and *KIF2C*, using constructs with either the 3’-UTR of the wild-type *KIF2C* (wt-KIF2C) or mutated *KIF2C* (mut-KIF2C). A schematic illustration depicts the putative binding site for *miR-4484* within the 3‘-UTR of wt-KIF2C and mut-KIF2C (Figure 4(A)). The wild-type sequence perfectly complements the *miR-4484* seed region, with -GCCUUU- in wt-*KIF2C* and -CGGAAA- in *miR-4484*. In contrast, the mutated sequence disrupts this interaction by replacing complementary bases with -AATGGC- in mut-KIF2C. The Dual-Luciferase Reporter Assay showed that cells transfected with the *miR-4484* mimic and the wild-type *KIF2C* 3‘-UTR (mimic+wt) exhibited a significant reduction in luciferase activity compared to the negative control (NC) group (Figure 4(B), *p <* 0.0001). This finding confirms that *miR-4484* directly binds to *KIF2C*. Additionally, it represses *KIF2C* expression. In contrast, cells transfected with the *miR-4484* mimic and the mutant *KIF2C* 3’-UTR (mimic+mut) exhibited luciferase activity comparable to the NC group (*p =* 0.2502), indicating that the mutation in *KIF2C* abolished *miR-4484* binding and repression. These results validate the direct interaction between *miR-4484* and the *KIF2C* 3’−UTR. They also confirm the specificity of this regulatory mechanism.

### KIF2C is essential for miR-4484-mediated regulation of HCC progression

**Figure 5.**
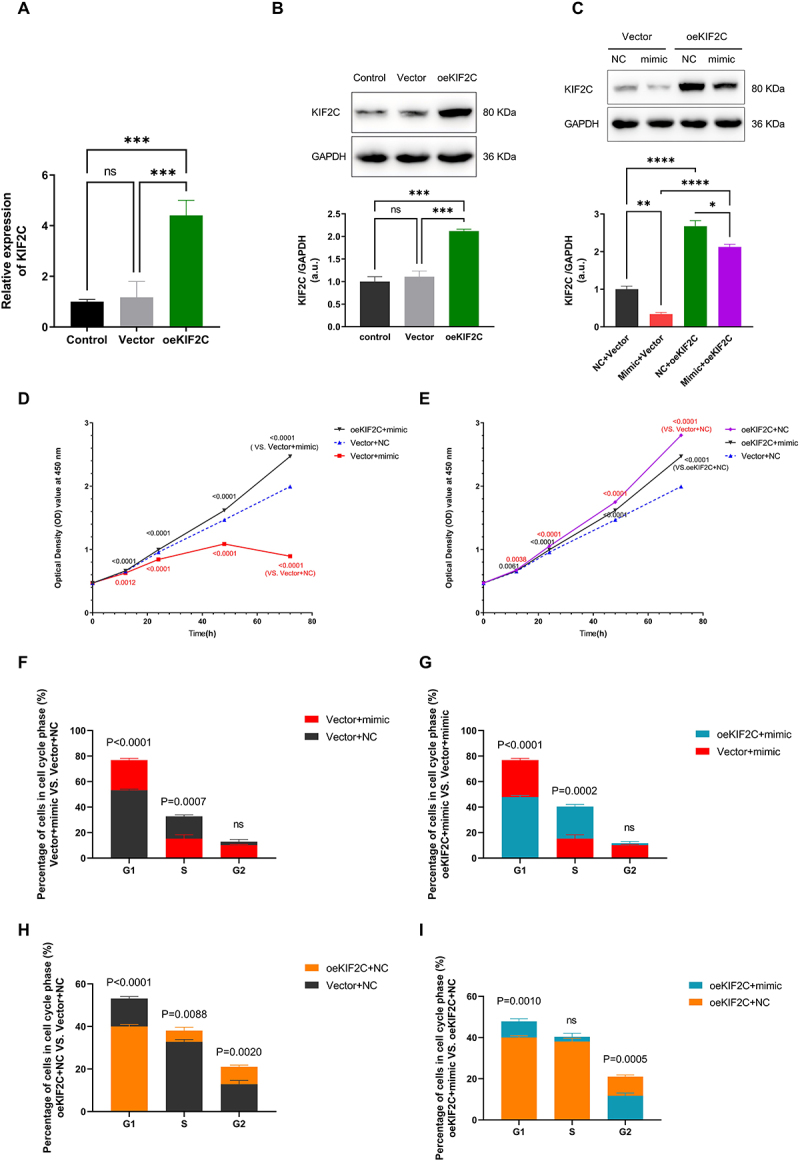
*miR-4484* regulates proliferation and cell cycle progression in HCC in a *KIF2C*-dependent manner. (A) *KIF2C* mRNA expression was analyzed in Huh7 cells transfected with either an empty vector (vector) or a *KIF2C* overexpression plasmid (oeKIF2C) using qRT-PCR. A total of 100 pg of RNA was reverse transcribed and then analyzed using qPCR with SYBR green and specific primers. *n* = 3 per group. GAPDH served as the normalization control. oeKIF2C significantly increased *KIF2C* levels compared to control and vector groups (*p <* 0.001). (B) KIF2C protein expression was analyzed in Huh7 cells transfected with either an empty vector (vector) or a KIF2C overexpression plasmid (oeKIF2C) using WB, with GAPDH as the internal control. All experiments were performed in triplicate. The KIF2C/GAPDH ratios were calculated. The values were then normalized to the average value of the control group. The results confirmed robust KIF2C protein over-expressed in the oeKIF2C group (*p <* 0.001), with no significant differences between control and vector groups. (C) KIF2C protein levels in Huh7 cells co-transfected with either a KIF2C plasmid (oeKIF2C) or NC plasmid (vector), and either a *miR-4484* mimic (mimic) or miR-NC (NC). All experiments were performed in triplicate. The KIF2C/GAPDH ratios were calculated. The values were then normalized to the average value of the vector+NC group. The expression level of KIF2C protein was lowest in the vector+mimic group, followed by the vector+NC group, with a significant difference between the two (*p <* 0.01). The oeKIF2C+NC group exhibited the highest KIF2C protein expression, whereas the oeKIF2C+mimic group showed a significant reduction compared with oeKIF2C+NC (*p <* 0.05). (D) the CCK-8 assay demonstrated that the *miR-4484* mimic (vector+mimic) significantly reduced cell viability compared to the vector+NC group (*p <* 0.01). KIF2C overexpression (oeKif2c+mimic) restored cell viability, surpassing levels seen in the vector+NC group (*p <* 0.0001). (E) the effects of KIF2C overexpression on cell proliferation were further examined. The oeKIF2C+NC group showed significantly increased proliferation compared to vector+NC at all time points (*p <* 0.01). In cases of KIF2C overexpression, treatment with the *miR-4484* mimic (oeKif2c+mimic) decreased cell proliferation compared to oeKIF2C+NC (*p <* 0.01). (F) Flow cytometry analysis revealed that *miR-4484* mimic treatment (vector+mimic) significantly increased the G1 phase population compared to vector+NC (*p <* 0.0001), accompanied by a reduction in the S phase proportion (*p =* 0.0007). No significant changes were observed in the G2 phase (ns), indicating G1 phase arrest. (G) When KIF2C was overexpressed (oeKif2c+mimic), the accumulation of the G1 phase induced by the *miR-4484* mimic was completely reversed (*p <* 0.0001), leading to a restoration of the S phase proportion (*p =* 0.0002). (H) KIF2C overexpression alone (oeKIF2C+NC) significantly decreased the G1 phase proportion (*p <* 0.0001) and increased both the S (*p =* 0.0088) and G2 phase populations (*p =* 0.0020) compared to the vector+NC group, highlighting KIF2C’s role in facilitating cell cycle progression. (I) the effects of the *miR-4484* mimic were further investigated by comparing the oeKIF2C+mimic group with the oeKIF2C+NC group in detail. *miR-4484* mimic (oeKIF2C+mimic) significantly increased the G1 phase proportion (*p =* 0.0010) and reduced the G2 phase proportion (*p =* 0.0005), while no significant changes were observed in the S phase (ns).

To validate the effect of the oeKIF2C plasmid, we transfected Huh7 cells with oeKIF2C or blank vector. The results were analysed using qRT-PCR (Figure 5(A)) and WB (Figure 5(B)). Both mRNA and protein levels of KIF2C were significantly elevated in the oeKIF2C group compared to the Control and Vector groups (*p <* 0.001). In contrast, no significant difference was observed between the Control and Vector groups, confirming the successful overexpression of KIF2C. Co-transfection experiments with oeKIF2C or blank vector along with either the *miR-4484* mimic or miR-NC resulted in four experimental groups: vector+NC, oeKIF2C+NC, vector+mimic, and oeKIF2C+mimic. WB results revealed that KIF2C protein expression was lowest in the vector+mimic group, moderate in the vector+NC group, and highest in the oeKIF2C+NC group. In the oeKIF2C+mimic group, KIF2C protein levels were slightly lower than those in the oeKIF2C+NC group, yet still significantly higher than in the vector+mimic and vector+NC groups (Figure 5(C)). These results demonstrate that the *miR-4484* mimic suppresses *KIF2C* expression. However, KIF2C overexpression overrides this effect, although there is still a slight reduction when the mimic is present.

The CCK-8 assay demonstrated that the *miR-4484* mimic (Vector+mimic) significantly reduced cell viability compared to the Vector+NC group (*p <* 0.01). KIF2C overexpression (oeKIF2C+mimic) restored cell viability, surpassing levels seen in the Vector+NC group (*p <* 0.0001), effectively reversing the *miR-4484* mimic’s inhibitory effect (Figure 5(D)). The effects of KIF2C overexpression on cell proliferation were further examined. The oeKIF2C+NC group showed significantly increased proliferation compared to Vector+NC at all time points (*p <* 0.01). In cases of KIF2C overexpression, treatment with the *miR-4484* mimic (oeKIF2C+mimic) decreased cell proliferation compared to oeKIF2C+NC (*p <* 0.01), suggesting that *miR-4484* partially inhibits the pro-proliferative effects of KIF2C (Figure 5(E)).

Flow cytometry analysis revealed that *miR-4484* mimic treatment (Vector+mimic) significantly increased the G1 phase population compared to Vector+NC (*p <* 0.0001), accompanied by a reduction in the S phase proportion (*p =* 0.0007). No significant changes were observed in the G2 phase (ns), indicating G1 phase arrest (Figure 5(F)). When KIF2C was overexpressed (oeKIF2C+mimic), the accumulation of the G1 phase induced by the *miR-4484* mimic was completely reversed (*p <* 0.0001), leading to a restoration of the S phase proportion (*p =* 0.0002) (Figure 5(G)). Furthermore, KIF2C overexpression alone (oeKIF2C+NC) significantly decreased the G1 phase proportion (*p <* 0.0001) and increased both the S (*p =* 0.0088) and G2 phase populations (*p =* 0.0020) compared to the Vector+NC group, highlighting KIF2C’s role in facilitating cell cycle progression (Figure 5(H)). The effects of the *miR-4484* mimic were further investigated by comparing the oeKIF2C+mimic group with the oeKIF2C+NC group in detail. *miR-4484* mimic (oeKIF2C+mimic) significantly increased the G1 phase proportion (*p =* 0.0010) and reduced the G2 phase proportion (*p =* 0.0005), while no significant changes were observed in the S phase (ns) (Figure 5(I)).

These findings collectively indicate that *miR-4484* suppresses HCC progression by inhibiting *KIF2C* expression, reducing proliferation, and inducing G1 phase arrest. However, these inhibitory effects are partially reversed by KIF2C overexpression, underscoring the functional interplay between *miR-4484* and *KIF2C* in regulating HCC progression.

### miR-4484 mimic represses tumour growth in vivo

**Figure 6.**
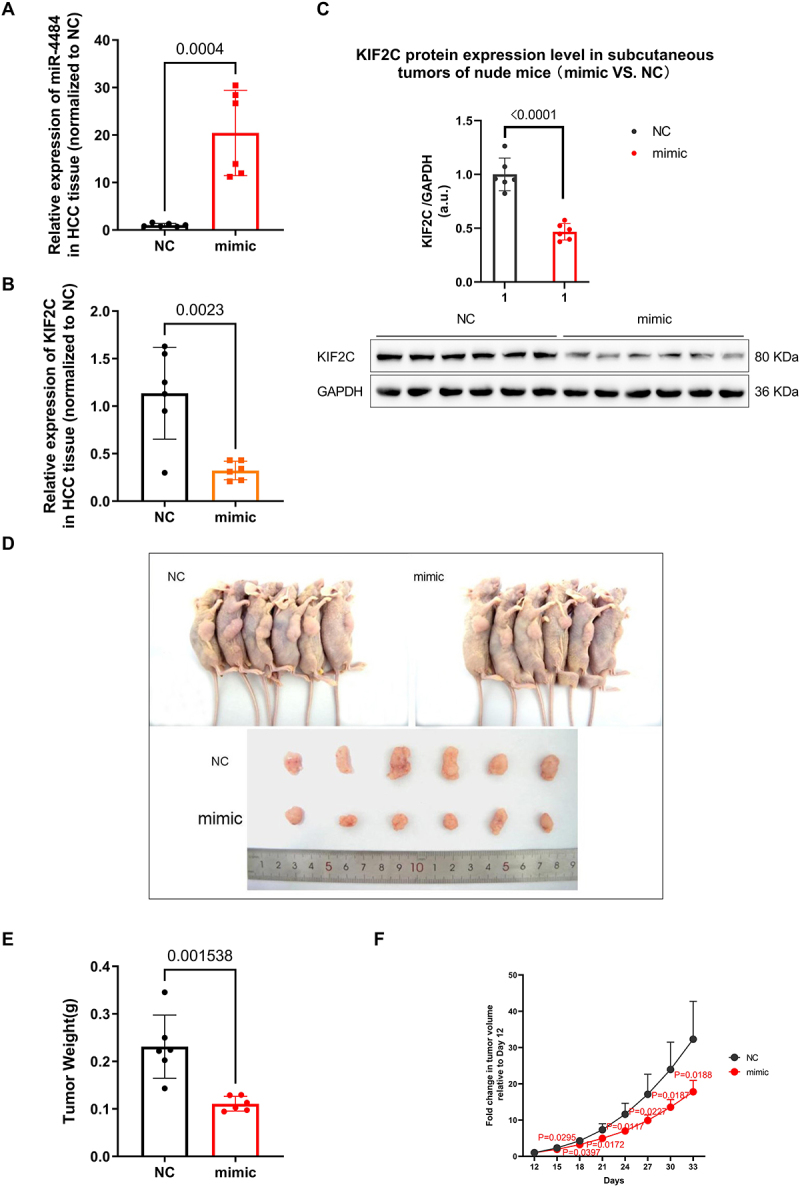
*miR-4484* mimic inhibits tumour growth in HCC *in vivo*. (A) the expression levels of miR-4484 in HCC tissues from tumour-bearing nude mice were measured with qRT-PCR. n = 6 per group. U6 was used as the normalization control. miR-4484 expression was significantly higher in the mimic group (injected with the miR-4484 mimic) compared to the NC group (injected with miR-NC) (*p* = 0.0004). (B)*KIF2C* mRNA expression in HCC tissues from tumor-bearing nude mice was evaluated using qRT-PCR. n = 6 per group. *GAPDH* served as the normalization control. *KIF2C* expression was significantly lower in the mimic group compared to the NC group (*p =* 0.0023). (C) KIF2C protein expression in HCC tissues from tumor-bearing nude mice was assessed using Western blot. n = 6 per group. GAPDH was used as the normalization control. KIF2C protein levels were significantly reduced in the mimic group compared to the NC group (*p <* 0.0001). (D) Representative images of subcutaneous tumors in nude mice. Tumors from the NC group are displayed in the upper-left panel, while tumors from the mimic group are shown in the upper-right panel. The lower panel presents excised tumors for direct comparison. (E) Tumor weights were measured post-sacrifice. Tumors from the mimic group were significantly lighter than those from the NC group (*p =* 0.0015).(F) the tumor growth curves compare the Fold change in tumor volume relative to Day 12 between the NC (negative control) and mimic groups. Tumor volumes were normalized to their respective volumes on Day 12, and Fold changes were calculated for each time point. The statistical significance between groups at each time point was assessed, with the corresponding *p* values indicated above the relevant points. From Day 15 onwards, the mimic group shows a significantly slower tumour growth rate than the NC group (*p <* 0.05).

To confirm the inhibitory effect of *miR-4484* on HCC progression, we repeatedly injected nude mice with xenograft tumours either with *miR-4484* mimic or miR-NC. A pathologist confirmed HCC in all subcutaneous tumour samples through histopathological examination using HE staining. Tumour samples were collected on Day 33 post-inoculation for further analysis. qRT-PCR analysis revealed a significant upregulation of *miR-4484* expression in the mimic group (Figure 6(A), *p =* 0.0004). Correspondingly, there was a downregulation of *KIF2C* mRNA (Figure 6(B), *p =* 0.0023) and protein levels (Figure 6(C), *p <* 0.0001). Representative images of subcutaneous tumours were captured after sacrifice (Figure 6(D)), revealing visibly smaller tumours in the mimic group compared to the miR-NC group. Quantitative analysis indicated that the mimic group had a significantly lower tumour weight (Figure 6(E), *p =* 0.0015).

Additionally, we monitored tumour growth starting on Day 12, measuring tumour dimensions every three days to calculate the tumour volume. While treatment also began on Day 12, significant differences in baseline tumour volumes were observed between the mimic and NC groups at this time point. This discrepancy was likely due to variations among individual animals or minor deviations during randomization. To address this, tumour growth data were normalized to the respective volumes on Day 12, enabling a more accurate evaluation of treatment effects. The fold change in tumour volume relative to Day 12 was calculated for each subsequent time point. From Day 15 onwards, the mimic group demonstrated a significantly slower tumour growth rate compared to the NC group (Figure 6(F), *p <* 0.05). These findings collectively demonstrate that *miR-4484* effectively suppresses HCC tumour progression *in vivo*.

## Discussion

HCC is a malignancy with poor prognosis, primarily due to its insidious onset and delayed diagnosis. Identifying specific miRNA markers for HCC is critical for improving early detection and therapeutic strategies. miRNAs play pivotal roles in HCC pathogenesis, influencing tumour proliferation, invasion, and metastasis [24]. Among these, *miR-4484* has emerged as a promising candidate for its tumour-suppressive properties.

In our study, we demonstrated that *miR-4484* expression were markedly reduced in HCC tissues and cell lines compared to normal liver tissues/cell line, as evidenced by analyse of the TCGA-LIHC database and *in vitro* experiments. Notably, lower *miR-4484* expression was correlated with worse survival outcomes in HCC patients, suggesting its role as a tumour suppressor. Importantly, the stability and presence of *miR-4484* in circulating body fluids make it an attractive candidate for developing non-invasive biomarkers. Tumour cells release miRNAs into the bloodstream through exosomes, apoptotic bodies, or passive leakage. Therefore, changes in circulating *miR-4484* levels may indicate tumour burden or aggressiveness, making it a useful tool for monitoring HCC progression and enabling early diagnosis. Consistent with our findings, Devis Pascut et al. also reported reduced serum *miR-4484* levels in HCC, further supporting its potential as a non-invasive biomarker for disease detection and surveillance [19].

As shown in Figure 1(E,F), the results revealed no significant differences in *miR-4484* expression across different N or M stages of HCC, suggesting that *miR-4484* primarily regulates tumour cell proliferation rather than invasion or metastasis. This is consistent with the high proliferative capacity of HCC and its relatively lower distant metastatic potential compared to cholangiocarcinoma. Based on this observation, our study prioritized proliferation assays, though we acknowledge the limitation of not investigating *miR-4484*‘s potential role in invasion or metastasis. Future studies will incorporate matrigel invasion assays, scratch assays, and *in vivo* metastasis models to comprehensively assess *miR-4484*‘s function in HCC progression.

To explore the downstream mechanisms of *miR-4484*, we employed TargetScan, miRTarBase, and miRWalk, identifying 10 overlapping predicted target genes. Among them, *KIF2C* emerged as a particularly well-studied oncogene in HCC, leading us to select it as a key downstream target of *miR-4484*. Further investigation revealed an inverse correlation between *miR-4484* and *KIF2C* expression, which was functionally validated through gain- and loss-of-function experiments. Overexpression of *miR-4484* downregulated *KIF2C*, suppressed tumour cell proliferation, and induced G1 phase arrest by inhibiting the G1-to-S phase transition. Conversely, *miR-4484* inhibition upregulated *KIF2C*, promoting cell proliferation and cell cycle progression. Dual-Luciferase reporter assays confirmed that *miR-4484* directly targets *KIF2C*, while rescue experiments and *in vivo* studies further substantiated this regulatory interaction.

Interestingly, while the regulatory effect of *miR-4484* on KIF2C mRNA and protein was consistently negative, differences were observed between KIF2C mRNA and protein expression levels. As shown in Figure 3(H), the expression level of *KIF2C* mRNA in the *miR-4484* inhibitor group was nearly three times that of the inhibitor-NC group. However, the protein expression level of KIF2C exhibited only a 1.7-fold increase (Figure 3(I)). This discrepancy can be attributed to several factors. While the *miR-4484* inhibitor significantly upregulated KIF2C expression at both the mRNA and protein levels, the magnitude of the change at the protein level was less pronounced compared to the mRNA level. Such inconsistencies are not unexpected and may arise from post-transcriptional regulation, variations in translation efficiency, or differences in protein stability. Additionally, factors such as protein turnover and the involvement of other regulatory pathways within the tumour microenvironment could contribute to this variation. Despite these variations in magnitude, the consistent directional changes in both mRNA and protein levels reinforce *miR-4484*‘s regulatory role in suppressing KIF2C and its oncogenic effects.

Moreover, our findings confirmed the tumour-suppressive role of *miR-4484*, as evidenced by its down-regulation in poorly differentiated tumours (G2, G3&G4) compared to G1. This suggests that *miR-4484* loss may contribute to tumour dedifferentiation and enhanced malignancy. In contrast, *miR-4484* expression was unexpectedly higher in advanced-stage tumours (T3&T4) or in cases with more aggressive pathology, creating a contradiction. We proposed several possible explanations: First, tumour cells may up-regulate *miR-4484* as a compensatory mechanism to counteract aggressive disease progression. Secondly, late-stage tumours exhibit greater cellular diversity, potentially including subpopulations with elevated *miR-4484* expression. Finally, even though *miR-4484* levels increase in advanced tumours, this internal response may not be strong enough to effectively suppress tumours. Consistently, *in vivo* xenograft experiments showed that direct *miR-4484* mimic injection significantly reduced tumour volume and weight, confirming its potent anti-tumour activity when exogenously restored.

Our study indicates that *miR-4484* acts as a tumour suppressor in HCC by targeting *KIF2C*, although the detailed mechanisms require further investigation. Prior studies have identified *KIF2C* as a key regulator linking the Wnt/β-catenin and mTORC1 pathways in HCC [25]. Activation of the Wnt/βcatenin pathway not only directly induces *KIF2C* expression but also promotes transcription of classical cellcycle regulators such as Cyclin D1 and Myc by preventing βcatenin degradation [25]. In addition, *KIF2C* serves as a central hub activating multiple oncogenic cascades, including PI3K/AKT/MAPK [24], which enhances mTORC1 signalling to drive protein synthesis, lipid and nucleotide metabolism, and inhibit autophagy – and Ras/MAPK [26], which facilitates tumour proliferation, migration, and invasion. Based on our findings, we propose that *miR‑4484* directly binds to *KIF2C* mRNA, leading to its degradation and reduced oncogenic activity. Conversely, downregulation of *miR‑4484* would result in *KIF2C* overexpression, thereby amplifying several oncogenic pathways and contributing to HCC progression. Collectively, these results highlight *miR‑4484* as both a potential prognostic biomarker and a promising therapeutic target in HCC.

While we confirmed the *miR-4484* – *KIF2C* interaction *in vitro* and *in vivo*, the broader molecular mechanisms remain unclear. *miR-4484* may also regulate other pathways in addition to *KIF2C*. Further exploration of these pathways and their interactions is necessary. Furthermore, while cell lines and mouse models provide useful information, they may not adequately reflect the complexity of human HCC. Larger clinical sample cohorts are needed to validate the prognostic and therapeutic potential of *miR-4484* in actual practice. Additionally, turning *miR-4484* into a therapeutic agent poses several challenges, especially regarding effective systemic delivery. miRNA mimics face obstacles such as: degradation by nucleases, rapid clearance, and potential off-target effects. Overcoming these hurdles requires innovative delivery systems capable of maintaining stability, tissue-specific targeting, and intracellular uptake [27]. New delivery platforms, such as lipid nanoparticles, exosomes, and viral vectors, show promise but require further evaluation for safety, efficacy, and immunogenicity [28]. Additionally, chemically modified miRNAs, such as locked nucleic acids or ligand-conjugated constructs, may enhance stability and targeting specificity [29–31]. Future research should prioritize the development of such delivery systems to facilitate the clinical application of *miR-4484*-based therapies.

In summary, *miR-4484* represents a promising biomarker and therapeutic target in HCC. Its regulatory role in *KIF2C*-mediated oncogenic pathways highlights its potential for clinical application, though further studies are warranted to fully elucidate its molecular functions and optimize its therapeutic utility.

## Conclusion

**Figure 7.**
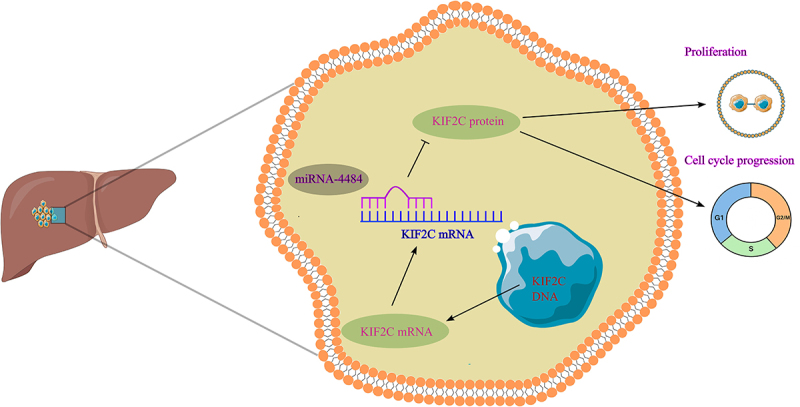
*miR-4484* suppresses cell proliferation and cell cycle progression by targeting *KIF2C* in HCC. *miR-4484* specifically targets the *KIF2C* mRNA, leading to its degradation and a subsequent reduction in KIF2C protein levels, thereby suppressing HCC proliferation and hindering cell cycle progression.

As illustrated in Figure 7, *miR-4484* suppresses HCC progression by targeting *KIF2C*, and its downregulation predicts poor prognosis, suggesting that further exploration of *miR-4484* may pave the way for novel biomarker development and therapeutic strategies in HCC.

## Supplementary Material

Supplementary tables.docx

Table S1 miR4484 inhibitor mimics sequence.docx

Table S4 KIF2C plasmid vetor construct.docx

Table S2 primers for qPCR.docx

Table S5 miR4484 expression.xlsx

Table S10 miRTarBase.xlsx

Table S7 miR4484 expr and pathological stages.docx

Table S11 miR4484 target gene predicted venn.xlsx

Table S8 TargetScan predicted targets.xlsx

Table S12 KIF2C expression.xlsx

Table S3 KIF2C sequence.docx

Table S13 expression of both miR4484 and KIF2C .xlsx

Table S9 mirwalk.xlsx

Table S6 miR4484 expression and OS.xlsx

## Data Availability

The datasets presented in this study can be found in online repositories or supplementary materials. The names of the repository/repositories and accession number(s) can be found in the article/supplementary materials.

## Abbreviations

ATCC: The American Type Culture Collection
CCK-8: Cell Counting Kit-8
CDS: Coding sequence
HCC: Hepatocellular carcinoma
HE: Haematoxylin and eosin
*KIF2C*: Kinesin family member 2C
LIHC: Liver Hepatocellular Carcinoma
MCAK: Mitotic centromere-associated kinesin
miRNAs: MicroRNAs
NCBI: The National Center for Biotechnology Information
OD: Oligonucleotide dose
Opti-MEM: Optimized Minimum Essential Medium
PLB: Passive Lysis Buffer
PVDF: Polyvinylidene fluoride
RIPA: Radioimmunoprecipitation assay
SDS-PAGE: Sodium dodecyl sulphate-polyacrylamide gel electrophoresis
SD: Standard Deviation
TCGA: The Cancer Genome Atlas database
