# Supplementary material for: miR-4484 suppresses hepatocellular carcinoma progression via targeting KIF2C
Source: RNA Biol. 2025 Oct 2;22(1):1–20. doi: 10.1080/15476286.2025.2569192 (PMC12498537; doi:10.1080/15476286.2025.2569192)
Supplement: Supplementary tables.docx [file KRNB_A_2569192_SM1642.docx]

**MiR-4484 suppresses hepatocellular carcinoma progression via targeting KIF2C**

Jianyang Lin, Yun Cai, Zhihong Chen, Jichun Ma, Kun Zhao

**Content of Supplementary Tables**

[Table S1: The RNA sequence of miR-4484, miR-4484 mimic, miR-4484 inhibitor.............................2](#bookmark1)

[Table S2: The related primers in qRT-PCR............................................................................................2](#bookmark2)

[Table S3: The sequence of wtKIF2C and mutKIF2C.............................................................................3](#bookmark3)

[Table S4: KIF2C plasmid vetor construct..............................................................................................4](#bookmark4)

[Table S5: The expression level of miR-4484 in TCGA-LIHC database................................................4](#bookmark5)

[Table S6: miR-4484 expression and overall survival data of HCC patients in TCGA-LIHC](#bookmark6) [database......................................................................................................................................................10](#bookmark6)

[Table S7: Statistical analysis of miR-4484 expression level and in the different pathological](#bookmark7) [subgroups...................................................................................................................................................15](#bookmark7)

[Table S8: Predicted target genes of miR-4484 from TargetScan database...........................................15](#bookmark8)

[Table S9: Predicted target genes of miR-4484 from miRWalk database.............................................1](#bookmark9)8

[Table S10: Predicted target genes of miR-4484 from miRTarBase database......................................19](#bookmark10)

[Table S11: Venn diagram analysis revealed 10 common target genes.................................................20](#bookmark11)

[Table S12: The expression level of KIF2C in TCGA-LIHC database.................................................21](#bookmark12)

[Table S13 Co-expression data of miR-4484 and KIF2C in TCGA-LIHC database........2](#bookmark13)9

**Table S1: The RNA sequence of miR-4484, miR-4484 mimic, miR-4484 inhibitor**

| **>hsa-miR-4484 MIMAT0019018** |
| --- |
| 5’-AAAAGGCGGGAGAAGCCCCA-3 ’ |
| **hsa-miR-4484 mimics sequence:** |
| 5’-AAAAGGCGGGAGAAGCCCCA-3 ’ |
| **hsa-miR-4484 Inhibitor sequence:** |
| 5’-UGGGGCUUCUCCCGCCUUUU-3 ’ |
| **miR-4484 N.C sequence：** |
| 5’-UUCUCCGAACGUGUCACGUTT-3 ’ |

**Table S2: The related primers in qRT-PCR**

(1) reverse-transcription primer:

5'-GTCGTATCCAGTGCAGGGTCCGAGGTATTCGCACTGGATACGACTGGGGC-3';

(2) miR-4484

Forward: 5'-GCGAAAAGGCGGGAGAA-3';

Reverse: 5'-AGTGCAGGGTCCGAGGTATT-3';

(3) U6 small nuclear 1 (RNU6-1)

Forward: 5'-CTCGCTTCGGCAGCACA-3'

Reverse: 5'-AACGCTTCACGAATTTGCGT-3';

(4) KIF2C

Forward: 5'-CCCAAGCTTATGGCCATGGACTCGTCG-3';

Reverse: 5'-CGGAATTCTCACTGGGGCCGTTTCTTG-3';

(5) GAPDH

Forward: 5'-AATCCCATCACCATCTTC-3';

Reverse: 5'-AGGCTGTTGTCATACTTC-3'.

**Table S3: The sequence of wtKIF2C and mutKIF2C**

**1.The wtKIF2C 3'-UTR is as followed:**

CGACTGCAAATAAAAATCTGTTTGGTTTGACACCCAGCCTCTTCCCTGGCCCTCC CCAGAGAACTTTGGGTACCTGGTGGGTCTAGGCAGGGTCTGAGCTGGGACAGGT TCTGGTAAATGCCAAGTATGGGGGCATCTGGGCCCAGGGCAGCTGGGGAGGGGG TCAGAGTGACATGGGACACTCCTTTTCTGTTCCTCAGTTGTCGCCCTCACGAGAG GAAGGAGCTCTTAGTTACCCTTTTGTGTTGCCCTTCTTTCCATCAAGGGGAATGTT CTCAGCATAGAGCTTTCTCCGCAGCATCCTGCCTGCGTGGACTGGCTGCTAATGG AGAGCTCCCTGGGGTTGTCCTGGCTCTGGGGAGAGAGACGGA*GCCTTT*AGTACA GCTATCTGCTGGCTCTAAACCTTCTAC*GCCTTT*GGGCCGAGCACTGAATGTCTTGT ACTTTAAAAAAATGTTTCTGAGACCTCTTTCTACTTTACTGTCTCCCTAGAGATCC TAGAGGATCCCTACTGTTTTCTGTTTTATGTGTTTATACATTGTATGTAACAATAA AGAGAAAAAATAAATCAGCTGTTTAAGTGTGTGGAAAAAAAAAAAAAAAAAA

**2.The mutKIF2C 3'-UTR is as followed:**

CGACTGCAAATAAAAATCTGTTTGGTTTGACACCCAGCCTCTTCCCTGGCCCTCC CCAGAGAACTTTGGGTACCTGGTGGGTCTAGGCAGGGTCTGAGCTGGGACAGGT TCTGGTAAATGCCAAGTATGGGGGCATCTGGGCCCAGGGCAGCTGGGGAGGGGG TCAGAGTGACATGGGACACTCCTTTTCTGTTCCTCAGTTGTCGCCCTCACGAGAG GAAGGAGCTCTTAGTTACCCTTTTGTGTTGCCCTTCTTTCCATCAAGGGGAATGTT CTCAGCATAGAGCTTTCTCCGCAGCATCCTGCCTGCGTGGACTGGCTGCTAATGG AGAGCTCCCTGGGGTTGTCCTGGCTCTGGGGAGAGAGACGGA*AATGGC*AGTACA GCTATCTGCTGGCTCTAAACCTTCTAT*AATGGC*GGGCCGAGCACTGAATGTCTTGT ACTTTAAAAAAATGTTTCTGAGACCTCTTTCTACTTTACTGTCTCCCTAGAGATCC TAGAGGATCCCTACTGTTTTCTGTTTTATGTGTTTATACATTGTATGTAACAATAA AGAGAAAAAATAAATCAGCTGTTTAAGTGTGTGGAAAAAAAAAAAAAAAAAA

Note: The italic sequence with underline and yellow background represents the combing region.

**Table S4: KIF2C plasmid vetor construct**


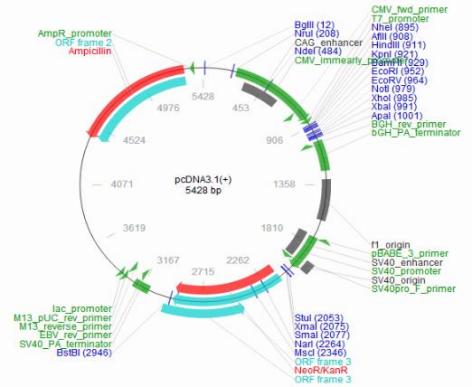


map of pCDNA3. 1(+) vector

KIF2C-F：5’-CCCAAGCTTATGGCCATGGACTCGTCG-3 ’ (Hind III) KIF2C-R：5’-CGGAATTCTCACTGGGGCCGTTTCTTG-3 ’ (EcoR I)

**Table S5: The expression level of miR-4484 in TCGA-LIHC database**

| miRNA_ID | Tissue type | hsa-mir-4484 |
| --- | --- | --- |
| TCGA-DD-A73D-01 | Tumor | 0.183024016 |
| TCGA-DD-A1EL-01 | Tumor | 0.548138684 |
| TCGA-ED-A8O5-01 | Tumor | 0.394734727 |
| TCGA-WX-AA47-01 | Tumor | 1.534825092 |
| TCGA-G3-A3CK-01 | Tumor | 0.29363416 |
| TCGA-RC-A7SB-01 | Tumor | 0.543586946 |
| TCGA-G3-A5SJ-01 | Tumor | 0.419019967 |
| TCGA-HP-A5MZ-01 | Tumor | 0.307303778 |
| TCGA-G3-A25Z-01 | Tumor | 0.248212987 |
| TCGA-DD-AADC-01 | Tumor | 0.260236473 |
| TCGA-DD-AACX-01 | Tumor | 0.837286919 |
| TCGA-NI-A8LF-01 | Tumor | 1.04521928 |
| TCGA-DD-AADQ-01 | Tumor | 0.608705176 |
| TCGA-DD-A115-01 | Tumor | 0.698886098 |
| TCGA-DD-A39W-01 | Tumor | 0.918241194 |
| TCGA-G3-A3CG-01 | Tumor | 1.891954946 |
| TCGA-5C-A9VG-01 | Tumor | 0.537443187 |
| TCGA-DD-AACN-01 | Tumor | 0.586543789 |
| TCGA-G3-A5SI-01 | Tumor | 0.609439161 |
| TCGA-DD-A11B-01 | Tumor | 0.363799053 |
| TCGA-DD-AADW-01 | Tumor | 0.38205076 |
| TCGA-XR-A8TF-01 | Tumor | 0.684781941 |
| TCGA-MI-A75G-01 | Tumor | 1.686776154 |
| TCGA-ED-A7XO-01 | Tumor | 1.606684789 |
| TCGA-DD-AACA-02B | Tumor | 0.70149849 |
| TCGA-CC-A8HT-01 | Tumor | 0.747022114 |
| TCGA-DD-AADS-01 | Tumor | 0.206474441 |
| TCGA-MR-A8JO-01 | Tumor | 0.700325186 |
| TCGA-BC-A10Q-11 | Normal | 1.239965598 |
| TCGA-FV-A3R2-11 | Normal | 0.653695672 |
| TCGA-MR-A520-01 | Tumor | 1.102139407 |
| TCGA-G3-A3CH-01 | Tumor | 1.160238037 |
| TCGA-DD-A1EL-11 | Normal | 0.522953638 |
| TCGA-DD-AAEE-01 | Tumor | 0.860417787 |
| TCGA-BC-A10W-11 | Normal | 0.726513876 |
| TCGA-ZS-A9CG-01 | Tumor | 0.900551916 |
| TCGA-BC-A10X-11 | Normal | 0.696408751 |
| TCGA-ZP-A9D1-01 | Tumor | 0.237552705 |
| TCGA-ZP-A9D4-01 | Tumor | 0.748058424 |
| TCGA-DD-A39W-11 | Normal | 0.929188045 |
| TCGA-WQ-A9G7-01 | Tumor | 1.184107605 |
| TCGA-G3-A5SK-01 | Tumor | 0.412562598 |
| TCGA-DD-AAEI-01 | Tumor | 0.40287495 |
| TCGA-DD-AACH-01 | Tumor | 0.144896069 |
| TCGA-FV-A3I0-11 | Normal | 0.219123283 |
| TCGA-DD-AAD2-01 | Tumor | 0.302617357 |
| TCGA-DD-AAW0-01 | Tumor | 0.414883483 |
| TCGA-G3-A7M9-01 | Tumor | 0.528873328 |
| TCGA-2Y-A9H7-01 | Tumor | 0.857996914 |
| TCGA-DD-A11B-11 | Normal | 1.218545607 |
| TCGA-DD-A11D-01 | Tumor | 1.121625515 |
| TCGA-DD-A11D-11 | Normal | 0.611267769 |
| TCGA-K7-AAU7-01 | Tumor | 0.863186785 |
| TCGA-DD-AAD1-01 | Tumor | 0.452339237 |
| TCGA-G3-A25Y-01 | Tumor | 0.739202633 |
| TCGA-CC-A9FU-01 | Tumor | 0.39443402 |
| TCGA-DD-A4NI-01 | Tumor | 0.647156069 |
| TCGA-CC-5264-01 | Tumor | 0.682339571 |
| TCGA-KR-A7K8-01 | Tumor | 1.214188233 |
| TCGA-DD-AACF-01 | Tumor | 0.550525417 |
| TCGA-DD-A3A3-11 | Normal | 0.674214758 |
| TCGA-CC-A3MA-01 | Tumor | 0.389887253 |
| TCGA-DD-AACB-01 | Tumor | 0.280621427 |
| TCGA-BC-A3KG-01 | Tumor | 2.632354775 |
| TCGA-DD-AAEK-01 | Tumor | 0.485499991 |
| TCGA-KR-A7K7-01 | Tumor | 1.2442039 |
| TCGA-O8-A75V-01 | Tumor | 0.368008395 |
| TCGA-DD-A118-11 | Normal | 1.39487189 |
| TCGA-DD-A1EA-01 | Tumor | 1.226462289 |
| TCGA-BC-A10T-11 | Normal | 0.329050096 |
| TCGA-DD-A3A1-11 | Normal | 0.497725784 |
| TCGA-G3-A6UC-01 | Tumor | 0.701789449 |
| TCGA-CC-A3M9-01 | Tumor | 0.275659094 |
| TCGA-3K-AAZ8-01 | Tumor | 0.676003763 |
| TCGA-DD-AADR-01 | Tumor | 1.619249598 |
| TCGA-EP-A12J-01 | Tumor | 0.254960414 |
| TCGA-ED-A82E-01 | Tumor | 0.623653391 |
| TCGA-DD-A3A4-11 | Normal | 0.396197858 |
| TCGA-RC-A6M6-01 | Tumor | 0.331236279 |
| TCGA-DD-A3A0-01 | Tumor | 0.417489079 |
| TCGA-DD-AACE-01 | Tumor | 0.805684551 |
| TCGA-RC-A7SK-01 | Tumor | 0.475385845 |
| TCGA-UB-A7MB-01 | Tumor | 0.818649355 |
| TCGA-QA-A7B7-01 | Tumor | 0.311682133 |
| TCGA-EP-A26S-11 | Normal | 0.919802281 |
| TCGA-UB-A7ME-01 | Tumor | 0.462407319 |
| TCGA-LG-A6GG-01 | Tumor | 0.539595587 |
| TCGA-BW-A5NQ-01 | Tumor | 1.433368647 |
| TCGA-DD-AADU-01 | Tumor | 1.312359643 |
| TCGA-DD-A1EH-01 | Tumor | 1.136378439 |
| TCGA-DD-A1EH-11 | Normal | 0.78572379 |
| TCGA-G3-A3CJ-01 | Tumor | 0.373778486 |
| TCGA-BD-A3EP-01 | Tumor | 0.297001197 |
| TCGA-BC-A10Z-01 | Tumor | 0.762875192 |
| TCGA-DD-AACS-01 | Tumor | 0.227068236 |
| TCGA-DD-A3A5-01 | Tumor | 0.214538899 |
| TCGA-G3-AAV1-01 | Tumor | 0.380268422 |
| TCGA-DD-AACI-01 | Tumor | 0.21547732 |
| TCGA-BC-A8YO-01 | Tumor | 0.311247337 |
| TCGA-BC-4072-01 | Tumor | 0.615699723 |
| TCGA-DD-A1EJ-11 | Normal | 0.225413146 |
| TCGA-BC-A216-01 | Tumor | 1.926140264 |
| TCGA-DD-A11A-11 | Normal | 1.112673447 |
| TCGA-DD-AADM-01 | Tumor | 0.247695449 |
| TCGA-BC-A216-11 | Normal | 1.015760879 |
| TCGA-BC-A5W4-01 | Tumor | 0.320711101 |
| TCGA-DD-A3A7-01 | Tumor | 0.296873196 |
| TCGA-DD-A1EE-01 | Tumor | 0.299269279 |
| TCGA-DD-A1EG-11 | Normal | 0.737775873 |
| TCGA-DD-A1EG-01 | Tumor | 0.204054303 |
| TCGA-RC-A7SF-01 | Tumor | 0.341816142 |
| TCGA-DD-AACW-01 | Tumor | 0.33589938 |
| TCGA-DD-A3A8-01 | Tumor | 0.85363882 |
| TCGA-BC-A217-01 | Tumor | 0.192543905 |
| TCGA-DD-A3A8-11 | Normal | 0.94385983 |
| TCGA-2Y-A9H0-01 | Tumor | 0.827419079 |
| TCGA-5R-AAAM-01 | Tumor | 0.275681737 |
| TCGA-BC-A10S-01 | Tumor | 0.390406854 |
| TCGA-DD-AAED-01 | Tumor | 0.292562678 |
| TCGA-BC-A10Y-01 | Tumor | 0.805868593 |
| TCGA-DD-A1EE-11 | Normal | 0.750432428 |
| TCGA-DD-A119-11 | Normal | 0.942804235 |
| TCGA-DD-AAE2-01 | Tumor | 0.82398816 |
| TCGA-DD-AADF-01 | Tumor | 0.654353963 |
| TCGA-FV-A3I1-01 | Tumor | 0.506493282 |
| TCGA-DD-AAVZ-01 | Tumor | 0.295199916 |
| TCGA-CC-5261-01 | Tumor | 0.394261689 |
| TCGA-DD-A1EB-11 | Normal | 1.116145807 |
| TCGA-DD-AACA-01 | Tumor | 0.297183198 |
| TCGA-DD-AACA-02 | Tumor | 0.677778828 |
| TCGA-FV-A4ZQ-01 | Tumor | 0.488050146 |
| TCGA-LG-A9QC-01 | Tumor | 0.325584441 |
| TCGA-FV-A23B-01 | Tumor | 0.662187266 |
| TCGA-FV-A23B-11 | Normal | 0.36420709 |
| TCGA-DD-AADL-01 | Tumor | 0.394530605 |
| TCGA-DD-A11C-11 | Normal | 1.615137525 |
| TCGA-DD-A11C-01 | Tumor | 1.13659762 |
| TCGA-DD-A4NP-01 | Tumor | 0.367245803 |
| TCGA-BC-A10Q-01 | Tumor | 0.998537087 |
| TCGA-BC-4073-01 | Tumor | 0.33402931 |
| TCGA-MI-A75E-01 | Tumor | 1.085160841 |
| TCGA-WQ-AB4B-01 | Tumor | 0.270002599 |
| TCGA-BC-A10U-01 | Tumor | 0.365461867 |
| TCGA-DD-AADA-01 | Tumor | 0.231464359 |
| TCGA-DD-A114-11 | Normal | 0.433613281 |
| TCGA-DD-A114-01 | Tumor | 0.313395629 |
| TCGA-DD-A3A6-01 | Tumor | 0.746102905 |
| TCGA-DD-A4NV-01 | Tumor | 0.871055864 |
| TCGA-DD-A3A6-11 | Normal | 0.341931112 |
| TCGA-ZP-A9CY-01 | Tumor | 0.84250905 |
| TCGA-DD-A1EC-01 | Tumor | 2.890241023 |
| TCGA-BC-A110-01 | Tumor | 0.421997198 |
| TCGA-BC-A110-11 | Normal | 1.50501387 |
| TCGA-FV-A3I1-11 | Normal | 1.031783345 |
| TCGA-DD-AACP-01 | Tumor | 0.268635602 |
| TCGA-DD-A1EC-11 | Normal | 1.008443861 |
| TCGA-DD-A1EB-01 | Tumor | 1.216302749 |
| TCGA-UB-A7MD-01 | Tumor | 0.237221053 |
| TCGA-RC-A6M3-01 | Tumor | 0.614789926 |
| TCGA-G3-A25T-01 | Tumor | 0.457080472 |
| TCGA-XR-A8TC-01 | Tumor | 0.616461213 |
| TCGA-K7-A5RG-01 | Tumor | 0.376647123 |
| TCGA-ES-A2HS-01 | Tumor | 0.480632213 |
| TCGA-DD-A39X-01 | Tumor | 0.389346536 |
| TCGA-ED-A7PX-01 | Tumor | 0.946784001 |
| TCGA-DD-AACO-01 | Tumor | 0.333948047 |
| TCGA-G3-A7M7-01 | Tumor | 0.884453351 |
| TCGA-DD-AAEH-01 | Tumor | 0.228391436 |
| TCGA-CC-5262-01 | Tumor | 0.529312229 |
| TCGA-2Y-A9H1-01 | Tumor | 0.346925128 |
| TCGA-BC-A10U-11 | Normal | 0.724168274 |
| TCGA-MI-A75I-01 | Tumor | 0.563073177 |
| TCGA-DD-AAE1-01 | Tumor | 0.499706616 |
| TCGA-G3-A3CH-11 | Normal | 0.26379763 |
| TCGA-DD-A1EI-11 | Normal | 1.864962638 |
| TCGA-CC-A7IE-01 | Tumor | 0.469001338 |
| TCGA-BC-A10Z-11 | Normal | 1.450854928 |
| TCGA-ZS-A9CE-01 | Tumor | 0.874662426 |
| TCGA-ED-A7PZ-01 | Tumor | 1.075963835 |
| TCGA-ED-A8O6-01 | Tumor | 0.422985374 |
| TCGA-2Y-A9H5-01 | Tumor | 0.45123802 |
| TCGA-BC-A10Y-11 | Normal | 0.612608221 |
| TCGA-CC-A3MB-01 | Tumor | 1.536766609 |
| TCGA-ZP-A9CZ-01 | Tumor | 0.379501195 |
| TCGA-2Y-A9GW-01 | Tumor | 1.009470438 |
| TCGA-BC-A10X-01 | Tumor | 0.464009495 |
| TCGA-DD-A39Z-01 | Tumor | 0.469578655 |
| TCGA-2Y-A9GT-01 | Tumor | 0.383496944 |
| TCGA-G3-AAV4-01 | Tumor | 0.512143939 |
| TCGA-BC-A3KF-01 | Tumor | 0.734633758 |
| TCGA-EP-A3JL-01 | Tumor | 0.225985612 |
| TCGA-BD-A2L6-11 | Normal | 0.261374352 |
| TCGA-BD-A2L6-01 | Tumor | 1.069913847 |
| TCGA-DD-AAVU-01 | Tumor | 0.188678169 |
| TCGA-BC-A10R-11 | Normal | 0.879059545 |
| TCGA-DD-A4NB-01 | Tumor | 1.265895917 |
| TCGA-G3-AAV2-01 | Tumor | 0.781091085 |
| TCGA-DD-AACM-01 | Tumor | 0.27841422 |
| TCGA-DD-A1EF-01 | Tumor | 0.422930494 |
| TCGA-G3-A25W-01 | Tumor | 1.298983179 |
| TCGA-T1-A6J8-01 | Tumor | 0.230036965 |
| TCGA-CC-A3MC-01 | Tumor | 0.345375936 |
| TCGA-DD-AAD3-01 | Tumor | 0.162623833 |
| TCGA-DD-A1ED-01 | Tumor | 1.098346788 |
| TCGA-G3-AAV5-01 | Tumor | 0.698509211 |
| TCGA-DD-A39X-11 | Normal | 0.261722159 |
| TCGA-2Y-A9GU-01 | Tumor | 0.356854839 |
| TCGA-CC-A9FW-01 | Tumor | 0.872950866 |
| TCGA-WX-AA46-01 | Tumor | 1.354539611 |
| TCGA-2Y-A9H4-01 | Tumor | 0.30183461 |
| TCGA-ZS-A9CF-01 | Tumor | 1.137074586 |
| TCGA-DD-AACJ-01 | Tumor | 0.236943189 |
| TCGA-ED-A627-01 | Tumor | 0.361722327 |
| TCGA-DD-A1EK-01 | Tumor | 0.342858499 |
| TCGA-MI-A75C-01 | Tumor | 0.424451322 |
| TCGA-DD-A113-01 | Tumor | 0.376958225 |
| TCGA-DD-AAC9-01 | Tumor | 0.214504085 |
| TCGA-DD-AAEA-01 | Tumor | 0.52954812 |
| TCGA-UB-AA0U-01 | Tumor | 0.215049823 |
| TCGA-DD-AAE8-01 | Tumor | 0.193648124 |
| TCGA-CC-A9FV-01 | Tumor | 0.357238941 |
| TCGA-YA-A8S7-01 | Tumor | 0.235055484 |
| TCGA-DD-AACZ-01 | Tumor | 0.389561305 |
| TCGA-RC-A6M5-01 | Tumor | 0.199562421 |
| TCGA-G3-AAUZ-01 | Tumor | 0.536685557 |
| TCGA-CC-A7IF-01 | Tumor | 0.98458133 |
| TCGA-5R-AA1D-01 | Tumor | 1.643389835 |
| TCGA-CC-A123-01 | Tumor | 0.558756451 |
| TCGA-DD-AADY-01 | Tumor | 0.778443258 |
| TCGA-UB-AA0V-01 | Tumor | 0.532030636 |
| TCGA-DD-AAEB-01 | Tumor | 0.226434544 |
| TCGA-EP-A3RK-11 | Normal | 0.929603927 |
| TCGA-ZS-A9CF-02 | Tumor | 0.249425923 |
| TCGA-DD-A118-01 | Tumor | 0.602599404 |
| TCGA-ED-A97K-01 | Tumor | 0.318057639 |
| TCGA-DD-AAVQ-01 | Tumor | 0.309851383 |
| TCGA-HP-A5N0-01 | Tumor | 0.635574246 |
| TCGA-DD-AACU-01 | Tumor | 0.255536989 |
| TCGA-EP-A12J-11 | Normal | 0.719192204 |
| TCGA-DD-AAVV-01 | Tumor | 0.157768111 |
| TCGA-2Y-A9GS-01 | Tumor | 0.406726042 |
| TCGA-ZP-A9CV-01 | Tumor | 0.25067058 |
| TCGA-4R-AA8I-01 | Tumor | 1.088742708 |
| TCGA-DD-AADP-01 | Tumor | 0.251371289 |
| TCGA-ED-A7PY-01 | Tumor | 0.945445897 |
| TCGA-DD-A116-11 | Normal | 0.61805443 |
| TCGA-DD-A116-01 | Tumor | 0.650221379 |
| TCGA-NI-A4U2-01 | Tumor | 1.024479261 |
| TCGA-DD-AAE6-01 | Tumor | 0.293629452 |
| TCGA-DD-A3A4-01 | Tumor | 1.906234886 |
| TCGA-2Y-A9HB-01 | Tumor | 0.295650153 |
| TCGA-DD-A73A-01 | Tumor | 1.09406645 |
| TCGA-G3-A3CI-01 | Tumor | 1.353698174 |
| TCGA-EP-A26S-01 | Tumor | 0.838660577 |
| TCGA-CC-A7IL-01 | Tumor | 0.64138048 |
| TCGA-G3-A25S-01 | Tumor | 0.532211216 |
| TCGA-DD-AAEG-01 | Tumor | 0.272674196 |
| TCGA-G3-A25U-01 | Tumor | 1.874678556 |
| TCGA-DD-A39Y-01 | Tumor | 0.300251438 |
| TCGA-DD-AAE4-01 | Tumor | 0.553122574 |
| TCGA-DD-A4ND-01 | Tumor | 0.451361474 |
| TCGA-CC-5260-01 | Tumor | 0.444687207 |
| TCGA-2Y-A9GX-01 | Tumor | 0.856203296 |
| TCGA-BD-A3EP-11 | Normal | 0.442321444 |
| TCGA-ED-A7XP-01 | Tumor | 0.479193303 |
| TCGA-FV-A2QR-11 | Normal | 0.316462058 |
| TCGA-FV-A2QR-01 | Tumor | 0.560817651 |
| TCGA-DD-A3A5-11 | Normal | 1.250115106 |
| TCGA-DD-AACY-01 | Tumor | 0.288730632 |
| TCGA-DD-A39V-01 | Tumor | 0.189165433 |
| TCGA-DD-A39V-11 | Normal | 0.448135538 |
| TCGA-G3-A25V-01 | Tumor | 0.207389382 |
| TCGA-BW-A5NO-01 | Tumor | 0.581257716 |
| TCGA-2Y-A9H9-01 | Tumor | 1.075575757 |
| TCGA-RC-A7S9-01 | Tumor | 0.974449283 |
| TCGA-CC-A9FS-01 | Tumor | 1.180114754 |
| TCGA-EP-A3RK-01 | Tumor | 0.422216851 |
| TCGA-UB-A7MC-01 | Tumor | 0.60090341 |

**Table S6: miR-4484 expression and overall survival data of HCC patients in TCGA-LIHC database**

| **sample_ID** | **Tissue type** | **hsa-mir-4484** | | **cutoff** | **group** | **OS_event** | **OS_time**  **（day）** |
| --- | --- | --- | --- | --- | --- | --- | --- |
| TCGA-2Y-A9GS-01 | Tumor | 0.406726042 | 0.7628 | | Low | 1 | 724 |
| TCGA-2Y-A9GT-01 | Tumor | 0.383496944 | 0.7628 | | Low | 1 | 1624 |
| TCGA-2Y-A9GU-01 | Tumor | 0.356854839 | 0.7628 | | Low | 0 | 1939 |
| TCGA-2Y-A9GW-01 | Tumor | 1.009470438 | 0.7628 | | High | 1 | 1271 |
| TCGA-2Y-A9GX-01 | Tumor | 0.856203296 | 0.7628 | | High | 0 | 2442 |
| TCGA-2Y-A9H0-01 | Tumor | 0.827419079 | 0.7628 | | High | 0 | 3675 |
| TCGA-2Y-A9H1-01 | Tumor | 0.346925128 | 0.7628 | | Low | 1 | 1229 |
| TCGA-2Y-A9H4-01 | Tumor | 0.30183461 | 0.7628 | | Low | 0 | 1452 |
| TCGA-2Y-A9H5-01 | Tumor | 0.45123802 | 0.7628 | | Low | 1 | 555 |
| TCGA-2Y-A9H7-01 | Tumor | 0.857996914 | 0.7628 | | High | 0 | 1168 |
| TCGA-2Y-A9H9-01 | Tumor | 1.075575757 | 0.7628 | | High | 0 | 697 |
| TCGA-2Y-A9HB-01 | Tumor | 0.295650153 | 0.7628 | | Low | 0 | 260 |
| TCGA-3K-AAZ8-01 | Tumor | 0.676003763 | 0.7628 | | Low | 0 | 396 |
| TCGA-4R-AA8I-01 | Tumor | 1.088742708 | 0.7628 | | High | 1 | 262 |
| TCGA-5C-A9VG-01 | Tumor | 0.537443187 | 0.7628 | | Low | 0 | 328 |
| TCGA-5R-AA1D-01 | Tumor | 1.643389835 | 0.7628 | | High | 0 | 449 |
| TCGA-5R-AAAM-01 | Tumor | 0.275681737 | 0.7628 | | Low | 1 | 46 |
| TCGA-BC-4072-01 | Tumor | 0.615699723 | 0.7628 | | Low | 1 | 1490 |
| TCGA-BC-4073-01 | Tumor | 0.33402931 | 0.7628 | | Low | 0 | 849 |
| TCGA-BC-A10Q-01 | Tumor | 0.998537087 | 0.7628 | | High | 1 | 1135 |
| TCGA-BC-A10S-01 | Tumor | 0.390406854 | 0.7628 | | Low | 1 | 1423 |
| TCGA-BC-A10U-01 | Tumor | 0.365461867 | 0.7628 | | Low | 1 | 837 |
| TCGA-BC-A10X-01 | Tumor | 0.464009495 | 0.7628 | | Low | 1 | 770 |
| TCGA-BC-A10Y-01 | Tumor | 0.805868593 | 0.7628 | | High | 1 | 711 |
| TCGA-BC-A10Z-01 | Tumor | 0.762875192 | 0.7628 | | High | 1 | 34 |
| TCGA-BC-A110-01 | Tumor | 0.421997198 | 0.7628 | | Low | 1 | 2116 |
| TCGA-BC-A216-01 | Tumor | 1.926140264 | 0.7628 | | High | 0 | 1351 |
| TCGA-BC-A217-01 | Tumor | 0.192543905 | 0.7628 | | Low | 1 | 1397 |
| TCGA-BC-A3KF-01 | Tumor | 0.734633758 | 0.7628 | | Low | 0 | 8 |
| TCGA-BC-A3KG-01 | Tumor | 2.632354775 | 0.7628 | | High | 0 | 680 |
| TCGA-BC-A5W4-01 | Tumor | 0.320711101 | 0.7628 | | Low | 1 | 547 |
| TCGA-BC-A8YO-01 | Tumor | 0.311247337 | 0.7628 | | Low | 0 | 562 |
| TCGA-BD-A2L6-01 | Tumor | 1.069913847 | 0.7628 | | High | 0 | 1363 |
| TCGA-BD-A3EP-01 | Tumor | 0.297001197 | 0.7628 | | Low | 0 | 409 |
| TCGA-BW-A5NO-01 | Tumor | 0.581257716 | 0.7628 | | Low | 0 | 20 |
| TCGA-BW-A5NQ-01 | Tumor | 1.433368647 | 0.7628 | | High | 0 | 0 |
| TCGA-CC-5260-01 | Tumor | 0.444687207 | 0.7628 | | Low | 1 | 87 |
| TCGA-CC-5261-01 | Tumor | 0.394261689 | 0.7628 | | Low | 1 | 97 |
| TCGA-CC-5262-01 | Tumor | 0.529312229 | 0.7628 | | Low | 1 | 103 |
| TCGA-CC-5264-01 | Tumor | 0.682339571 | 0.7628 | | Low | 1 | 102 |
| TCGA-CC-A123-01 | Tumor | 0.558756451 | 0.7628 | | Low | 0 | 219 |
| TCGA-CC-A3M9-01 | Tumor | 0.275659094 | 0.7628 | | Low | 1 | 300 |
| TCGA-CC-A3MA-01 | Tumor | 0.389887253 | 0.7628 | | Low | 1 | 303 |
| TCGA-CC-A3MB-01 | Tumor | 1.536766609 | 0.7628 | | High | 1 | 315 |
| TCGA-CC-A3MC-01 | Tumor | 0.345375936 | 0.7628 | | Low | 0 | 363 |
| TCGA-CC-A7IE-01 | Tumor | 0.469001338 | 0.7628 | | Low | 1 | 217 |
| TCGA-CC-A7IF-01 | Tumor | 0.98458133 | 0.7628 | | High | 1 | 649 |
| TCGA-CC-A7IL-01 | Tumor | 0.64138048 | 0.7628 | | Low | 1 | 278 |
| TCGA-CC-A8HT-01 | Tumor | 0.747022114 | 0.7628 | | Low | 1 | 140 |
| TCGA-CC-A9FS-01 | Tumor | 1.180114754 | 0.7628 | | High | 0 | 211 |
| TCGA-CC-A9FU-01 | Tumor | 0.39443402 | 0.7628 | | Low | 0 | 0 |
| TCGA-CC-A9FV-01 | Tumor | 0.357238941 | 0.7628 | | Low | 0 | 0 |
| TCGA-CC-A9FW-01 | Tumor | 0.872950866 | 0.7628 | | High | 0 | 248 |
| TCGA-DD-A113-01 | Tumor | 0.376958225 | 0.7628 | | Low | 0 | 2425 |
| TCGA-DD-A114-01 | Tumor | 0.313395629 | 0.7628 | | Low | 1 | 1149 |
| TCGA-DD-A115-01 | Tumor | 0.698886098 | 0.7628 | | Low | 1 | 2542 |
| TCGA-DD-A116-01 | Tumor | 0.650221379 | 0.7628 | | Low | 1 | 1622 |
| TCGA-DD-A118-01 | Tumor | 0.602599404 | 0.7628 | | Low | 0 | 3437 |
| TCGA-DD-A11B-01 | Tumor | 0.363799053 | 0.7628 | | Low | 1 | 14 |
| TCGA-DD-A11C-01 | Tumor | 1.13659762 | 0.7628 | | High | 0 | 662 |
| TCGA-DD-A11D-01 | Tumor | 1.121625515 | 0.7628 | | High | 1 | 1560 |
| TCGA-DD-A1EA-01 | Tumor | 1.226462289 | 0.7628 | | High | 0 | 2415 |
| TCGA-DD-A1EB-01 | Tumor | 1.216302749 | 0.7628 | | High | 0 | 2017 |
| TCGA-DD-A1EC-01 | Tumor | 2.890241023 | 0.7628 | | High | 0 | 602 |
| TCGA-DD-A1ED-01 | Tumor | 1.098346788 | 0.7628 | | High | 0 | 2301 |
| TCGA-DD-A1EE-01 | Tumor | 0.299269279 | 0.7628 | | Low | 1 | 349 |
| TCGA-DD-A1EF-01 | Tumor | 0.422930494 | 0.7628 | | Low | 1 | 394 |
| TCGA-DD-A1EG-01 | Tumor | 0.204054303 | 0.7628 | | Low | 1 | 1372 |
| TCGA-DD-A1EH-01 | Tumor | 1.136378439 | 0.7628 | | High | 0 | 1495 |
| TCGA-DD-A1EK-01 | Tumor | 0.342858499 | 0.7628 | | Low | 1 | 558 |
| TCGA-DD-A1EL-01 | Tumor | 0.548138684 | 0.7628 | | Low | 1 | 415 |
| TCGA-DD-A39V-01 | Tumor | 0.189165433 | 0.7628 | | Low | 1 | 643 |
| TCGA-DD-A39W-01 | Tumor | 0.918241194 | 0.7628 | | High | 1 | 827 |
| TCGA-DD-A39X-01 | Tumor | 0.389346536 | 0.7628 | | Low | 1 | 1694 |
| TCGA-DD-A39Y-01 | Tumor | 0.300251438 | 0.7628 | | Low | 1 | 171 |
| TCGA-DD-A39Z-01 | Tumor | 0.469578655 | 0.7628 | | Low | 1 | 601 |
| TCGA-DD-A3A0-01 | Tumor | 0.417489079 | 0.7628 | | Low | 1 | 785 |
| TCGA-DD-A3A4-01 | Tumor | 1.906234886 | 0.7628 | | High | 1 | 612 |
| TCGA-DD-A3A5-01 | Tumor | 0.214538899 | 0.7628 | | Low | 1 | 3125 |
| TCGA-DD-A3A6-01 | Tumor | 0.746102905 | 0.7628 | | Low | 1 | 3258 |
| TCGA-DD-A3A7-01 | Tumor | 0.296873196 | 0.7628 | | Low | 1 | 419 |
| TCGA-DD-A3A8-01 | Tumor | 0.85363882 | 0.7628 | | High | 1 | 11 |
| TCGA-DD-A4NB-01 | Tumor | 1.265895917 | 0.7628 | | High | 0 | 989 |
| TCGA-DD-A4ND-01 | Tumor | 0.451361474 | 0.7628 | | Low | 0 | 2746 |
| TCGA-DD-A4NI-01 | Tumor | 0.647156069 | 0.7628 | | Low | 0 | 816 |
| TCGA-DD-A4NP-01 | Tumor | 0.367245803 | 0.7628 | | Low | 0 | 3308 |
| TCGA-DD-A4NV-01 | Tumor | 0.871055864 | 0.7628 | | High | 0 | 2398 |
| TCGA-DD-A73A-01 | Tumor | 1.09406645 | 0.7628 | | High | 0 | 728 |
| TCGA-DD-A73D-01 | Tumor | 0.183024016 | 0.7628 | | Low | 0 | 693 |
| TCGA-DD-AAC9-01 | Tumor | 0.214504085 | 0.7628 | | Low | 0 | 347 |
| TCGA-DD-AACA-01 | Tumor | 0.297183198 | 0.7628 | | Low | 0 | 2301 |
| TCGA-DD-AACA-02 | Tumor | 0.677778828 | 0.7628 | | Low | 0 | 2301 |
| TCGA-DD-AACB-01 | Tumor | 0.280621427 | 0.7628 | | Low | 0 | 2324 |
| TCGA-DD-AACE-01 | Tumor | 0.805684551 | 0.7628 | | High | 0 | 2184 |
| TCGA-DD-AACF-01 | Tumor | 0.550525417 | 0.7628 | | Low | 1 | 365 |
| TCGA-DD-AACH-01 | Tumor | 0.144896069 | 0.7628 | | Low | 1 | 195 |
| TCGA-DD-AACI-01 | Tumor | 0.21547732 | 0.7628 | | Low | 0 | 1618 |
| TCGA-DD-AACJ-01 | Tumor | 0.236943189 | 0.7628 | | Low | 0 | 2102 |
| TCGA-DD-AACM-01 | Tumor | 0.27841422 | 0.7628 | | Low | 0 | 1769 |
| TCGA-DD-AACN-01 | Tumor | 0.586543789 | 0.7628 | | Low | 0 | 1302 |
| TCGA-DD-AACO-01 | Tumor | 0.333948047 | 0.7628 | | Low | 0 | 1876 |
| TCGA-DD-AACP-01 | Tumor | 0.268635602 | 0.7628 | | Low | 0 | 415 |
| TCGA-DD-AACS-01 | Tumor | 0.227068236 | 0.7628 | | Low | 0 | 1804 |
| TCGA-DD-AACU-01 | Tumor | 0.255536989 | 0.7628 | | Low | 0 | 1567 |
| TCGA-DD-AACW-01 | Tumor | 0.33589938 | 0.7628 | | Low | 0 | 1424 |
| TCGA-DD-AACX-01 | Tumor | 0.837286919 | 0.7628 | | High | 0 | 170 |
| TCGA-DD-AACY-01 | Tumor | 0.288730632 | 0.7628 | | Low | 0 | 1450 |
| TCGA-DD-AACZ-01 | Tumor | 0.389561305 | 0.7628 | | Low | 1 | 171 |
| TCGA-DD-AAD1-01 | Tumor | 0.452339237 | 0.7628 | | Low | 0 | 564 |
| TCGA-DD-AAD2-01 | Tumor | 0.302617357 | 0.7628 | | Low | 0 | 658 |
| TCGA-DD-AAD3-01 | Tumor | 0.162623833 | 0.7628 | | Low | 0 | 1295 |
| TCGA-DD-AADA-01 | Tumor | 0.231464359 | 0.7628 | | Low | 0 | 1233 |
| TCGA-DD-AADC-01 | Tumor | 0.260236473 | 0.7628 | | Low | 1 | 425 |
| TCGA-DD-AADF-01 | Tumor | 0.654353963 | 0.7628 | | Low | 1 | 115 |
| TCGA-DD-AADL-01 | Tumor | 0.394530605 | 0.7628 | | Low | 0 | 636 |
| TCGA-DD-AADM-01 | Tumor | 0.247695449 | 0.7628 | | Low | 1 | 12 |
| TCGA-DD-AADP-01 | Tumor | 0.251371289 | 0.7628 | | Low | 0 | 458 |
| TCGA-DD-AADQ-01 | Tumor | 0.608705176 | 0.7628 | | Low | 0 | 436 |
| TCGA-DD-AADR-01 | Tumor | 1.619249598 | 0.7628 | | High | 0 | 2028 |
| TCGA-DD-AADS-01 | Tumor | 0.206474441 | 0.7628 | | Low | 0 | 474 |
| TCGA-DD-AADU-01 | Tumor | 1.312359643 | 0.7628 | | High | 0 | 554 |
| TCGA-DD-AADW-01 | Tumor | 0.38205076 | 0.7628 | | Low | 0 | 587 |
| TCGA-DD-AADY-01 | Tumor | 0.778443258 | 0.7628 | | High | 0 | 555 |
| TCGA-DD-AAE1-01 | Tumor | 0.499706616 | 0.7628 | | Low | 0 | 552 |
| TCGA-DD-AAE2-01 | Tumor | 0.82398816 | 0.7628 | | High | 0 | 638 |
| TCGA-DD-AAE4-01 | Tumor | 0.553122574 | 0.7628 | | Low | 0 | 608 |
| TCGA-DD-AAE6-01 | Tumor | 0.293629452 | 0.7628 | | Low | 0 | 141 |
| TCGA-DD-AAE8-01 | Tumor | 0.193648124 | 0.7628 | | Low | 0 | 664 |
| TCGA-DD-AAEA-01 | Tumor | 0.52954812 | 0.7628 | | Low | 0 | 575 |
| TCGA-DD-AAEB-01 | Tumor | 0.226434544 | 0.7628 | | Low | 0 | 478 |
| TCGA-DD-AAED-01 | Tumor | 0.292562678 | 0.7628 | | Low | 0 | 763 |
| TCGA-DD-AAEE-01 | Tumor | 0.860417787 | 0.7628 | | High | 0 | 810 |
| TCGA-DD-AAEG-01 | Tumor | 0.272674196 | 0.7628 | | Low | 0 | 719 |
| TCGA-DD-AAEH-01 | Tumor | 0.228391436 | 0.7628 | | Low | 0 | 784 |
| TCGA-DD-AAEI-01 | Tumor | 0.40287495 | 0.7628 | | Low | 0 | 1531 |
| TCGA-DD-AAEK-01 | Tumor | 0.485499991 | 0.7628 | | Low | 0 | 1067 |
| TCGA-DD-AAVQ-01 | Tumor | 0.309851383 | 0.7628 | | Low | 0 | 2728 |
| TCGA-DD-AAVU-01 | Tumor | 0.188678169 | 0.7628 | | Low | 0 | 2202 |
| TCGA-DD-AAVV-01 | Tumor | 0.157768111 | 0.7628 | | Low | 0 | 2455 |
| TCGA-DD-AAVZ-01 | Tumor | 0.295199916 | 0.7628 | | Low | 0 | 1900 |
| TCGA-DD-AAW0-01 | Tumor | 0.414883483 | 0.7628 | | Low | 0 | 2015 |
| TCGA-ED-A627-01 | Tumor | 0.361722327 | 0.7628 | | Low | 0 | 423 |
| TCGA-ED-A7PX-01 | Tumor | 0.946784001 | 0.7628 | | High | 0 | 6 |
| TCGA-ED-A7PY-01 | Tumor | 0.945445897 | 0.7628 | | High | 0 | 390 |
| TCGA-ED-A7PZ-01 | Tumor | 1.075963835 | 0.7628 | | High | 0 | 6 |
| TCGA-ED-A7XO-01 | Tumor | 1.606684789 | 0.7628 | | High | 0 | 427 |
| TCGA-ED-A7XP-01 | Tumor | 0.479193303 | 0.7628 | | Low | 0 | 400 |
| TCGA-ED-A82E-01 | Tumor | 0.623653391 | 0.7628 | | Low | 0 | 408 |
| TCGA-ED-A8O5-01 | Tumor | 0.394734727 | 0.7628 | | Low | 0 | 406 |
| TCGA-ED-A8O6-01 | Tumor | 0.422985374 | 0.7628 | | Low | 1 | 56 |
| TCGA-ED-A97K-01 | Tumor | 0.318057639 | 0.7628 | | Low | 0 | 6 |
| TCGA-EP-A12J-01 | Tumor | 0.254960414 | 0.7628 | | Low | 0 | 570 |
| TCGA-EP-A26S-01 | Tumor | 0.838660577 | 0.7628 | | High | 0 | 608 |
| TCGA-EP-A3JL-01 | Tumor | 0.225985612 | 0.7628 | | Low | 0 | 303 |
| TCGA-EP-A3RK-01 | Tumor | 0.422216851 | 0.7628 | | Low | 0 | 363 |
| TCGA-ES-A2HS-01 | Tumor | 0.480632213 | 0.7628 | | Low | 1 | 688 |
| TCGA-FV-A23B-01 | Tumor | 0.662187266 | 0.7628 | | Low | 1 | 1852 |
| TCGA-FV-A2QR-01 | Tumor | 0.560817651 | 0.7628 | | Low | 1 | 581 |
| TCGA-FV-A3I1-01 | Tumor | 0.506493282 | 0.7628 | | Low | 1 | 247 |
| TCGA-FV-A4ZQ-01 | Tumor | 0.488050146 | 0.7628 | | Low | 0 | 12 |
| TCGA-G3-A25S-01 | Tumor | 0.532211216 | 0.7628 | | Low | 1 | 416 |
| TCGA-G3-A25T-01 | Tumor | 0.457080472 | 0.7628 | | Low | 0 | 1553 |
| TCGA-G3-A25U-01 | Tumor | 1.874678556 | 0.7628 | | High | 0 | 1636 |
| TCGA-G3-A25V-01 | Tumor | 0.207389382 | 0.7628 | | Low | 0 | 860 |
| TCGA-G3-A25W-01 | Tumor | 1.298983179 | 0.7628 | | High | 0 | 935 |
| TCGA-G3-A25Y-01 | Tumor | 0.739202633 | 0.7628 | | Low | 1 | 452 |
| TCGA-G3-A25Z-01 | Tumor | 0.248212987 | 0.7628 | | Low | 0 | 655 |
| TCGA-G3-A3CG-01 | Tumor | 1.891954946 | 0.7628 | | High | 0 | 673 |
| TCGA-G3-A3CH-01 | Tumor | 1.160238037 | 0.7628 | | High | 0 | 780 |
| TCGA-G3-A3CI-01 | Tumor | 1.353698174 | 0.7628 | | High | 0 | 180 |
| TCGA-G3-A3CJ-01 | Tumor | 0.373778486 | 0.7628 | | Low | 0 | 594 |
| TCGA-G3-A3CK-01 | Tumor | 0.29363416 | 0.7628 | | Low | 0 | 585 |
| TCGA-G3-A5SI-01 | Tumor | 0.609439161 | 0.7628 | | Low | 1 | 768 |
| TCGA-G3-A5SJ-01 | Tumor | 0.419019967 | 0.7628 | | Low | 0 | 698 |
| TCGA-G3-A5SK-01 | Tumor | 0.412562598 | 0.7628 | | Low | 0 | 744 |
| TCGA-G3-A6UC-01 | Tumor | 0.701789449 | 0.7628 | | Low | 0 | 671 |
| TCGA-G3-A7M7-01 | Tumor | 0.884453351 | 0.7628 | | High | 0 | 361 |
| TCGA-G3-A7M9-01 | Tumor | 0.528873328 | 0.7628 | | Low | 1 | 56 |
| TCGA-G3-AAUZ-01 | Tumor | 0.536685557 | 0.7628 | | Low | 0 | 480 |
| TCGA-G3-AAV1-01 | Tumor | 0.380268422 | 0.7628 | | Low | 1 | 359 |
| TCGA-G3-AAV2-01 | Tumor | 0.781091085 | 0.7628 | | High | 0 | 372 |
| TCGA-G3-AAV4-01 | Tumor | 0.512143939 | 0.7628 | | Low | 1 | 27 |
| TCGA-G3-AAV5-01 | Tumor | 0.698509211 | 0.7628 | | Low | 0 | 354 |
| TCGA-HP-A5MZ-01 | Tumor | 0.307303778 | 0.7628 | | Low | 1 | 91 |
| TCGA-HP-A5N0-01 | Tumor | 0.635574246 | 0.7628 | | Low | 1 | 1147 |
| TCGA-K7-A5RG-01 | Tumor | 0.376647123 | 0.7628 | | Low | 0 | 519 |
| TCGA-K7-AAU7-01 | Tumor | 0.863186785 | 0.7628 | | High | 0 | 359 |
| TCGA-KR-A7K7-01 | Tumor | 1.2442039 | 0.7628 | | High | 0 | 951 |
| TCGA-KR-A7K8-01 | Tumor | 1.214188233 | 0.7628 | | High | 0 | 906 |
| TCGA-LG-A6GG-01 | Tumor | 0.539595587 | 0.7628 | | Low | 0 | 387 |
| TCGA-LG-A9QC-01 | Tumor | 0.325584441 | 0.7628 | | Low | 0 | 425 |
| TCGA-MI-A75C-01 | Tumor | 0.424451322 | 0.7628 | | Low | 0 | 291 |
| TCGA-MI-A75E-01 | Tumor | 1.085160841 | 0.7628 | | High | 0 | 507 |
| TCGA-MI-A75G-01 | Tumor | 1.686776154 | 0.7628 | | High | 0 | 698 |
| TCGA-MI-A75I-01 | Tumor | 0.563073177 | 0.7628 | | Low | 0 | 630 |
| TCGA-MR-A520-01 | Tumor | 1.102139407 | 0.7628 | | High | 0 | 229 |
| TCGA-MR-A8JO-01 | Tumor | 0.700325186 | 0.7628 | | Low | 0 | 330 |
| TCGA-NI-A4U2-01 | Tumor | 1.024479261 | 0.7628 | | High | 1 | 1791 |
| TCGA-NI-A8LF-01 | Tumor | 1.04521928 | 0.7628 | | High | 0 | 799 |
| TCGA-O8-A75V-01 | Tumor | 0.368008395 | 0.7628 | | Low | 0 | 538 |
| TCGA-QA-A7B7-01 | Tumor | 0.311682133 | 0.7628 | | Low | 0 | 94 |
| TCGA-RC-A6M3-01 | Tumor | 0.614789926 | 0.7628 | | Low | 0 | 0 |
| TCGA-RC-A6M5-01 | Tumor | 0.199562421 | 0.7628 | | Low | 0 | 15 |
| TCGA-RC-A6M6-01 | Tumor | 0.331236279 | 0.7628 | | Low | 0 | 9 |
| TCGA-RC-A7S9-01 | Tumor | 0.974449283 | 0.7628 | | High | 0 | 640 |
| TCGA-RC-A7SB-01 | Tumor | 0.543586946 | 0.7628 | | Low | 0 | 588 |
| TCGA-RC-A7SF-01 | Tumor | 0.341816142 | 0.7628 | | Low | 0 | 579 |
| TCGA-RC-A7SK-01 | Tumor | 0.475385845 | 0.7628 | | Low | 0 | 472 |
| TCGA-T1-A6J8-01 | Tumor | 0.230036965 | 0.7628 | | Low | 0 | 23 |
| TCGA-UB-A7MB-01 | Tumor | 0.818649355 | 0.7628 | | High | 0 | 601 |
| TCGA-UB-A7MC-01 | Tumor | 0.60090341 | 0.7628 | | Low | 0 | 500 |
| TCGA-UB-A7MD-01 | Tumor | 0.237221053 | 0.7628 | | Low | 1 | 52 |
| TCGA-UB-A7ME-01 | Tumor | 0.462407319 | 0.7628 | | Low | 0 | 486 |
| TCGA-UB-AA0U-01 | Tumor | 0.215049823 | 0.7628 | | Low | 0 | 327 |
| TCGA-UB-AA0V-01 | Tumor | 0.532030636 | 0.7628 | | Low | 0 | 314 |
| TCGA-WQ-A9G7-01 | Tumor | 1.184107605 | 0.7628 | | High | 0 | 30 |
| TCGA-WQ-AB4B-01 | Tumor | 0.270002599 | 0.7628 | | Low | 0 | 395 |
| TCGA-WX-AA46-01 | Tumor | 1.354539611 | 0.7628 | | High | 0 | 756 |
| TCGA-WX-AA47-01 | Tumor | 1.534825092 | 0.7628 | | High | 1 | 556 |
| TCGA-XR-A8TC-01 | Tumor | 0.616461213 | 0.7628 | | Low | 0 | 1339 |
| TCGA-XR-A8TF-01 | Tumor | 0.684781941 | 0.7628 | | Low | 1 | 693 |
| TCGA-YA-A8S7-01 | Tumor | 0.235055484 | 0.7628 | | Low | 1 | 412 |
| TCGA-ZP-A9CV-01 | Tumor | 0.25067058 | 0.7628 | | Low | 1 | 1088 |
| TCGA-ZP-A9CY-01 | Tumor | 0.84250905 | 0.7628 | | High | 0 | 782 |
| TCGA-ZP-A9CZ-01 | Tumor | 0.379501195 | 0.7628 | | Low | 0 | 706 |
| TCGA-ZP-A9D1-01 | Tumor | 0.237552705 | 0.7628 | | Low | 0 | 21 |
| TCGA-ZP-A9D4-01 | Tumor | 0.748058424 | 0.7628 | | Low | 0 | 395 |
| TCGA-ZS-A9CE-01 | Tumor | 0.874662426 | 0.7628 | | High | 0 | 1241 |
| TCGA-ZS-A9CF-01 | Tumor | 1.137074586 | 0.7628 | | High | 0 | 2412 |
| TCGA-ZS-A9CF-02 | Tumor | 0.249425923 | 0.7628 | | Low | 0 | 2412 |
| TCGA-ZS-A9CG-01 | Tumor | 0.900551916 | 0.7628 | | High | 0 | 341 |

**Table S7: Statistical analysis of miR-4484 expression level and in the different pathological subgroups**

| Pathological type | Total  number | Group(n) | | | P-Value |
| --- | --- | --- | --- | --- | --- |
| Histologic_grade (Mean ± SD) | 226 | G1 (n = 33) | G2 (n = 109) | G3&G4 (n = 84) | 0.0279 |
|  |  | 0.72 ± 0.34 | 0.61 ± 0.40 | 0.61 ± 0.52 |  |
| T stage  (Mean ± SD) | 229 | T1 (n = 115) | T2 (n = 59) | T3&T4 (n = 55) | 0.0259 |
|  |  | 0.58 ± 0.42 | 0.66 ± 0.45 | 0.72 ± 0.44 |  |
| N stage  (Mean ± SD) | 228 | N0 (n = 44) | N1&NX  (n = 184) |  | 0.3047 |
|  |  | 0.55 ± 0.33 | 0.65 ± 0.46 |  |  |
| M stage  (Mean ± SD) | 229 | M0 (n = 167) | M1&Mx  (n = 62) |  | 0.316 |
|  |  | 0.63 ± 0.47 | 0.63 ± 0.34 |  |  |
| Pathologic stage (Mean ± SD) | 214 | Stage I (n = 107) | Stage II  (n = 54) | StageIII&StageIV  (n = 53) | 0.1114 |
|  |  | 0.59 ± 0.43 | 0.66 ± 0.46 | 0.7030 ± 0.4458 |  |
|  |  | Stage III & Stage IV VS. Stage I | | | 0.0307 |

**Table S8: Predicted target genes of miR-4484 from TargetScan database**

| MAP3K7CL | NACC1 | G3BP2 | ZNF672 |
| --- | --- | --- | --- |
| ZNF30 | SLC1A2 | MAT1A | WNT5A |
| HSD11B1L | ZNF639 | FUCA1 | MINOS1 |
| YBX1 | NPPB | AC069547.1 | TADA2B |
| USP13 | DDX17 | KCNK13 | COX17 |
| C10orf115 | AIP | FHL2 | RP11-1396O13.13 |
| ZNF737 | APIP | VAPA | ZNF627 |
| AC015987.2 | FETUB | TM4SF1 | RAB33B |
| NYAP2 | AC138655.1 | CC2D1B | GPALPP1 |
| RP11-834C11.12 | CCDC144NL | PTCH2 | FOXP1 |
| ZNF791 | RP11-386G21.2 | UBE2NL | MAGEA11 |
| ZNF616 | ANKRD39 | PSEN2 | MAP3K8 |
| NME4 | LDHB | TSN | FAXDC2 |
| SACM1L | DDAH1 | OSTC | ING5 |
| CTAGE15 | LRRC8B | CTGF | VAMP3 |
| CTAGE6 | C2orf83 | CPSF3L | FKRP |
| SHC2 | GABARAP | MLLT10 | GOLGA8O |
| ZNF107 | ZNF558 | POP5 | TBK1 |
| RP11-422N16.3 | UBA2 | HECTD3 | GOLGA8R |
| TAF13 | PDCD10 | CST3 | ZNF141 |
| STK32A | RP11-268J15.5 | PLP2 | TCF7 |
| HOXC5 | TMEM154 | SEC61B | MTMR12 |
| SOD3 | PDCD1LG2 | SATB2 | FAM175B |
| MRPL41 | FITM2 | ZIK1 | COX4I1 |
| CTAG1B | IGLON5 | SLC25A43 | EDA2R |
| CTAG1A | IQGAP1 | ARPP21 | SLC25A30 |
| LCMT2 | OTC | XG | ARID2 |
| ZNF479 | TFEB | ENAH | PIGM |
| AATF | AL009178.1 | PRR15L | BLZF1 |
| ZNF268 | TFPI2 | AC012215.1 | ZNF587B |
| MSRB3 | KRTAP5-4 | PBRM1 | ZNF845 |
| RAP2A | RABIF | MEMO1 | EFNA1 |
| SULT4A1 | RETSAT | EFNA2 | VPS52 |
| AP1S1 | RBPJ | SPRY2 | DIMT1 |
| HECTD2 | HES1 | TMEM179B | CD2BP2 |
| ADAM8 | APP | CLEC4M | SEH1L |
| B4GALT1 | BAIAP2L1 | MLF2 | PIM1 |
| RP11-318A15.7 | KRTAP5-1 | NABP1 | GOLGA8H |
| ZNF98 | TM9SF1 | GPR31 | RAPGEF2 |
| ANLN | NHLH1 | ZSCAN22 | GOLGA8K |
| IER3 | THAP4 | SNX30 | GOLGA8N |
| CCDC3 | MLIP | ERMN | SLC2A12 |
| LITAF | RAP2B | CALU | RASSF3 |
| FOXE1 | C2orf69 | SSR3 | ETV5 |
| OSTM1 | MCUR1 | PHPT1 | MAX |
| RECQL | CYP1B1 | RSL24D1 | ZNF486 |
| P2RY1 | CARM1 | C3orf72 | CXXC1 |
| SLCO1B3 | ARHGEF6 | ZNF675 | ZSCAN23 |
| RP11-1102P16.1 | HIST2H2BE | RIMKLB | PLCL1 |
| IQCK | EEPD1 | UBE2E3 | F8A1 |
| KIF22 | FAM20B | RAB3A | CLK3 |
| PSMG4 | ZNF492 | PDZD9 | PDYN |
| LRRC16A | ZNF677 | ZNF780B | CCSAP |
| GLUD2 | HIST2H4B | NUP214 | AP1G1 |
| R3HDM1 | TXN2 | TMEM128 | F8A2 |
| PPP1R18 | PEBP1 | TMEM26 | F8A3 |
| NGFRAP1 | TEAD1 | MYF6 | PAK7 |
| SPRYD4 | RNASEL | KLHL8 | C9orf72 |
| FSIP1 | PTP4A3 | C7orf65 | KM-PA-2 |
| EIF4A2 | BEST1 | TBX22 | LRRC45 |
| CTD-2140B24.4 | RIT2 | C8orf31 | RD3 |
| CFC1B | C17orf97 | APCDD1 | ENPEP |
| PPP1R14B | MBP | MFSD11 | ZNF835 |
| SKAP1 | EIF4E | HPS1 | TMEM62 |
| ZNF488 | SEPT11 | DDIT4L | GOLGA8M |
| CFC1 | GLUD1 | DUSP5 | PPP2R5C |
| EXD2 | ZNF821 | USP45 | GOLGA8I |
| PCGF3 | STC1 | SERINC5 | CLHC1 |
| OLFM2 | NUP160 | CIAO1 | C10orf25 |
| NR4A1 | STOM | FAM71E2 | PNRC2 |
| RRAGA | AADACL4 | NETO1 | HNRNPA1 |
| ZBTB47 | CBY1 | ZNF117 | ZNF77 |
| SELT | PCDHB3 | DAK | MVB12A |
| SKP2 | CTB-186H2.3 | CMKLR1 | TMEM64 |
| PSD3 | GPR12 | PPAP2A | TNFRSF9 |
| PABPN1 | SLCO2B1 | C9orf43 | ST3GAL3 |
| G6PC2 | HLA-DQA1 | GOLGA8J | C9orf62 |
| PCDHB4 | ATP5F1 | ZNF763 | MAP1LC3B |
| BHLHE40 | C16orf87 | WDR6 | PM20D2 |
| VAPB | ZMPSTE24 | MPL | RFTN2 |
| ATM | HIST2H4A | ZNF565 | GATAD2A |
| ATP5S | LRRC7 | BLCAP | EIF2AK1 |
| CC2D2A | GPR15 | PLEKHO2 | SELRC1 |
| SMG7 | IGF2R | CILP | BMP2K |
| PTDSS1 | TBCB | UBXN10 | RNPC3 |
| NPM1 | ENY2 | AMZ2 | NT5E |
| AL354808.2 | MMD | EMC10 | TMEM52B |
| ZNF302 | ITGB1 | C7orf41 | SPIN1 |
| CDK4 | REL | SLC25A36 | SH3TC2 |
| AK3 | CCT8L2 | AP4S1 | DLG1 |
| RAB11FIP2 | DOK5 | CCDC121 | FAIM3 |
| ZNF253 | ITFG1 | C3orf58 | INSM1 |
| BCL2L2-PABPN1 | SLC37A4 | NHLRC3 | RAD54B |
| ZNF772 | ZDHHC15 | PTPRA | ASCC1 |
| RHOU | ZNF582 | ZNF788 | HES7 |
| TXNDC2 | DOC2A | DBF4B | FUNDC2 |
| C9orf9 | GATA6 | MYADM | RAB3B |
| ISCA1 | ZNF416 | CYP4V2 | HS6ST2 |
| LAMB3 | CERS2 | ZMAT3 | BOP1 |
| SCARB1 | AXIN2 | ORC6 | DKFZP667F0711 |
| ZNF728 | UNG | GOSR2 | SLC28A1 |
| GRPEL2 | CD69 | CLEC4D | TAL1 |
| KIF2C | TMEM38A | TAF4 | CRCP |
| P2RY10 | IFNW1 | NRP2 | ZNF596 |
| TMED2 | BCKDHB | DKFZP434H0512 | TRMT13 |
| RBFOX2 | ARHGAP32 | AL353791.1 | SEC22C |
| ZNF792 | RFX6 | TET2 | RCAN2 |
| PPP2R3C | COX7B | URI1 | RPSAP58 |
| ZNF676 | GINS4 | FSHB | MAPK4 |
| SLC25A28 | KLHL42 | TP53INP1 | FOXD4L3 |
| BX088651.1 | PALM3 | MYLIP | SLC22A15 |
| GALNS | C2orf80 | PARM1 | FOXD4L6 |
| FRG1B | SPO11 | MOB1B | UBL3 |
| POLR1C | PVRL4 | ATP8B3 | SLC39A2 |
| CHDC2 | NIPAL3 | ACHE | ANGEL2 |
| MYLK4 | AC006372.1 | C12orf61 | DAG1 |
| ZFP69B | FBXO34 | LIX1L | PRPF38A |
| ANXA1 | SUCO | CKAP2 | POLR2C |
| SURF1 | NKIRAS1 | PLEKHH1 | DIRAS3 |
| C17orf96 | MRPS6 | ZNF267 | FAM20C |
| ZMYM6NB | MYCN | MORN4 | ZNF544 |
| FAM49B | PRR3 | PAX8 | ADAMTS20 |
| BPHL | SMAP2 | SGPP1 | ZNF667 |
| AFAP1 | MTHFD2 | CBX3 |  |
| ARPC3 | AGO3 | TMC7 |  |
| KRTAP4-2 | CDC5L | LRRC63 |  |
| ZNF395 | SLC17A2 | DEPTOR |  |
| CASC3 | ZNF696 | TP53TG3B |  |
| SGOL2 | RABAC1 | FBXO11 |  |
| EPGN | PARP9 | LRFN2 |  |
| C18orf25 | CHPF | PROSER2 |  |
| LRMP | ZNF705B | TP53TG3 |  |
| RAB31 | ZNF705D | TP53TG3D |  |
| ATOX1 | ZNF99 | TTPAL |  |
| EVI5 | FFAR3 | NCAM1 |  |
| ACP6 | DNAJC5B | ZNF697 |  |
| BRE | IDO2 | ZNF578 |  |
| RP11-362K2.2 | FAM83F | POLR3B |  |
| TMEM184B | KRT74 | TP53TG3C |  |
| PATL2 | PIK3C2A | DHX15 |  |

**Table S9: Predicted target genes of miR-4484 from miRWalk database**

| ZNF148 |
| --- |
| PLEKHA8 |
| PABPN1 |
| UCK2 |
| CDON |
| RBFOX2 |
| GATAD1 |
| PTPRF |
| CTC1 |
| AK4 |
| ZNF791 |
| GXYLT1 |
| CLCN3 |
| ARL5B |
| CERS2 |
| APP |
| ZMYND11 |
| XRCC3 |
| MAPKAPK5 |
| BCL2L2-PABPN1 |
| THSD4 |
| CADM1 |
| ZBTB7A |
| RETSAT |
| INO80D |
| RBM28 |
| SCNM1 |
| KPNB1 |
| CDC42SE1 |
| NDUFV3 |
| CENPO |
| SLC30A7 |
| PTCH2 |
| SERINC5 |
| LITAF |
| PTPN14 |
| KIF2C |
| GABARAP |
| HAVCR1 |
| LIMD1 |
| LAMC1 |
| PEBP1 |
| RBMS2 |
| RPL14 |
| CACNA2D2 |
| MZT1 |
| ANKRD23 |
| CYP1B1 |

**Table S10: Predicted target genes of miR-4484 from miRTarBase database**

| LAMC1 |
| --- |
| KIF2C |
| ZMYND11 |
| NPM1 |
| NDUFV3 |
| LIMD1 |
| SERINC5 |
| ZNF91 |
| THSD4 |
| HAVCR1 |
| CACNA2D2 |
| GABARAP |
| RBM28 |
| UCK2 |
| TAF13 |
| SLC30A7 |
| PTPRF |
| GXYLT1 |
| EFNA1 |
| LRRC45 |
| MZT1 |
| CYP1B1 |
| PEBP1 |
| CTC1 |
| KPNB1 |
| PTCH2 |
| LITAF |
| SDR42E1 |
| CDC42SE1 |
| CEP19 |
| CERS2 |
| P2RY1 |
| ZNF148 |
| PLEKHA8 |
| CADM1 |
| APIP |
| CDON |
| AK4 |
| RIPK4 |
| ZNF791 |
| PTPN14 |
| INO80D |
| ARL5B |
| XRCC3 |
| ZBTB7A |
| CLCN3 |
| SCNM1 |
| GATAD1 |
| SEPHS1 |
| RPL14 |
| RBMS2 |
| MAPKAPK5 |
| APP |
| Mmp21 |

**Table S11: Venn diagram analysis revealed 10 common target genes**

| KIF2C |
| --- |
| SERINC5 |
| GABARAP |
| CYP1B1 |
| PEBP1 |
| PTCH2 |
| LITAF |
| CERS2 |
| ZNF791 |
| APP |

**Table S12: The expression level of KIF2C in TCGA-LIHC database**

| sample_ID | Tissue type | KIF2C |
| --- | --- | --- |
| TCGA-CC-A7II-01 | Tumor | 11.2 |
| TCGA-CC-A3M9-01 | Tumor | 11.1 |
| TCGA-G3-A7M9-01 | Tumor | 11.09 |
| TCGA-BC-A10W-01 | Tumor | 10.77 |
| TCGA-BC-A216-01 | Tumor | 10.66 |
| TCGA-CC-A7IJ-01 | Tumor | 10.66 |
| TCGA-G3-AAV7-01 | Tumor | 10.54 |
| TCGA-CC-A8HT-01 | Tumor | 10.44 |
| TCGA-CC-A5UE-01 | Tumor | 10.41 |
| TCGA-BW-A5NQ-01 | Tumor | 10.39 |
| TCGA-CC-A1HT-01 | Tumor | 10.21 |
| TCGA-CC-5264-01 | Tumor | 10.17 |
| TCGA-DD-AACL-01 | Tumor | 10.16 |
| TCGA-DD-A39Y-01 | Tumor | 10.13 |
| TCGA-CC-5263-01 | Tumor | 10.02 |
| TCGA-CC-A7IG-01 | Tumor | 9.899 |
| TCGA-BC-A3KG-01 | Tumor | 9.878 |
| TCGA-2Y-A9GY-01 | Tumor | 9.835 |
| TCGA-RC-A6M6-01 | Tumor | 9.817 |
| TCGA-DD-A114-01 | Tumor | 9.805 |
| TCGA-QA-A7B7-01 | Tumor | 9.786 |
| TCGA-FV-A4ZP-01 | Tumor | 9.724 |
| TCGA-CC-A9FU-01 | Tumor | 9.695 |
| TCGA-DD-AACH-01 | Tumor | 9.668 |
| TCGA-CC-A3MA-01 | Tumor | 9.661 |
| TCGA-CC-A8HU-01 | Tumor | 9.647 |
| TCGA-BC-A69H-01 | Tumor | 9.631 |
| TCGA-RC-A6M3-01 | Tumor | 9.611 |
| TCGA-DD-AADC-01 | Tumor | 9.61 |
| TCGA-DD-AADN-01 | Tumor | 9.597 |
| TCGA-YA-A8S7-01 | Tumor | 9.579 |
| TCGA-BC-A8YO-01 | Tumor | 9.576 |
| TCGA-CC-A5UD-01 | Tumor | 9.56 |
| TCGA-2Y-A9H0-01 | Tumor | 9.53 |
| TCGA-DD-AADD-01 | Tumor | 9.491 |
| TCGA-DD-AACV-01 | Tumor | 9.446 |
| TCGA-DD-AACG-01 | Tumor | 9.438 |
| TCGA-DD-AACP-01 | Tumor | 9.411 |
| TCGA-G3-A5SI-01 | Tumor | 9.374 |
| TCGA-KR-A7K7-01 | Tumor | 9.339 |
| TCGA-5C-AAPD-01 | Tumor | 9.301 |
| TCGA-DD-AACZ-01 | Tumor | 9.296 |
| TCGA-BC-4073-01 | Tumor | 9.295 |
| TCGA-DD-A4NQ-01 | Tumor | 9.293 |
| TCGA-DD-AA3A-01 | Tumor | 9.293 |
| TCGA-G3-A25X-01 | Tumor | 9.272 |
| TCGA-RC-A7S9-01 | Tumor | 9.241 |
| TCGA-EP-A2KA-01 | Tumor | 9.227 |
| TCGA-WQ-A9G7-01 | Tumor | 9.225 |
| TCGA-DD-A4NE-01 | Tumor | 9.214 |
| TCGA-UB-A7MF-01 | Tumor | 9.177 |
| TCGA-CC-5258-01 | Tumor | 9.175 |
| TCGA-ZP-A9CZ-01 | Tumor | 9.165 |
| TCGA-ED-A8O6-01 | Tumor | 9.151 |
| TCGA-DD-A1EL-01 | Tumor | 9.121 |
| TCGA-CC-A7IE-01 | Tumor | 9.117 |
| TCGA-BC-4072-01 | Tumor | 9.111 |
| TCGA-CC-A5UC-01 | Tumor | 9.109 |
| TCGA-FV-A4ZQ-01 | Tumor | 9.106 |
| TCGA-BC-A217-01 | Tumor | 9.104 |
| TCGA-2Y-A9GS-01 | Tumor | 9.102 |
| TCGA-2Y-A9H2-01 | Tumor | 9.093 |
| TCGA-DD-AAD5-01 | Tumor | 9.063 |
| TCGA-DD-AAE0-01 | Tumor | 9.058 |
| TCGA-G3-A25T-01 | Tumor | 9.039 |
| TCGA-DD-A3A7-01 | Tumor | 9.028 |
| TCGA-DD-AAE6-01 | Tumor | 9.026 |
| TCGA-CC-A3MB-01 | Tumor | 8.995 |
| TCGA-RG-A7D4-01 | Tumor | 8.99 |
| TCGA-UB-A7MA-01 | Tumor | 8.99 |
| TCGA-BC-A10Y-01 | Tumor | 8.973 |
| TCGA-G3-A5SJ-01 | Tumor | 8.97 |
| TCGA-UB-A7MB-01 | Tumor | 8.955 |
| TCGA-ED-A459-01 | Tumor | 8.953 |
| TCGA-ZS-A9CF-01 | Tumor | 8.913 |
| TCGA-K7-A5RG-01 | Tumor | 8.904 |
| TCGA-CC-A7IK-01 | Tumor | 8.897 |
| TCGA-CC-A123-01 | Tumor | 8.852 |
| TCGA-DD-AACB-01 | Tumor | 8.848 |
| TCGA-G3-AAV6-01 | Tumor | 8.847 |
| TCGA-ED-A7XO-01 | Tumor | 8.828 |
| TCGA-DD-AADW-01 | Tumor | 8.811 |
| TCGA-DD-AAD6-01 | Tumor | 8.802 |
| TCGA-G3-A25S-01 | Tumor | 8.792 |
| TCGA-5C-A9VG-01 | Tumor | 8.786 |
| TCGA-DD-A1EJ-01 | Tumor | 8.745 |
| TCGA-2Y-A9H8-01 | Tumor | 8.745 |
| TCGA-2V-A95S-01 | Tumor | 8.743 |
| TCGA-ED-A7PZ-01 | Tumor | 8.74 |
| TCGA-CC-5262-01 | Tumor | 8.737 |
| TCGA-EP-A3RK-01 | Tumor | 8.73 |
| TCGA-K7-AAU7-01 | Tumor | 8.714 |
| TCGA-DD-AADV-01 | Tumor | 8.712 |
| TCGA-CC-A8HV-01 | Tumor | 8.695 |
| TCGA-XR-A8TD-01 | Tumor | 8.684 |
| TCGA-UB-AA0U-01 | Tumor | 8.671 |
| TCGA-CC-A8HS-01 | Tumor | 8.663 |
| TCGA-DD-AADB-01 | Tumor | 8.634 |
| TCGA-FV-A495-01 | Tumor | 8.633 |
| TCGA-DD-AACO-01 | Tumor | 8.616 |
| TCGA-DD-AADF-01 | Tumor | 8.599 |
| TCGA-CC-A3MC-01 | Tumor | 8.588 |
| TCGA-DD-AADO-01 | Tumor | 8.586 |
| TCGA-CC-5260-01 | Tumor | 8.566 |
| TCGA-DD-AACX-01 | Tumor | 8.547 |
| TCGA-DD-AAE4-01 | Tumor | 8.537 |
| TCGA-DD-AACC-01 | Tumor | 8.531 |
| TCGA-G3-A25Y-01 | Tumor | 8.528 |
| TCGA-ED-A7PX-01 | Tumor | 8.511 |
| TCGA-DD-A4NJ-01 | Tumor | 8.5 |
| TCGA-ED-A66Y-01 | Tumor | 8.498 |
| TCGA-MI-A75I-01 | Tumor | 8.476 |
| TCGA-BC-A112-01 | Tumor | 8.471 |
| TCGA-G3-A7M6-01 | Tumor | 8.426 |
| TCGA-DD-A4NR-01 | Tumor | 8.409 |
| TCGA-DD-AAEI-01 | Tumor | 8.401 |
| TCGA-DD-AADR-01 | Tumor | 8.392 |
| TCGA-DD-AAVQ-01 | Tumor | 8.346 |
| TCGA-G3-AAUZ-01 | Tumor | 8.332 |
| TCGA-XR-A8TG-01 | Tumor | 8.314 |
| TCGA-DD-AADM-01 | Tumor | 8.309 |
| TCGA-BC-A10Q-01 | Tumor | 8.299 |
| TCGA-BD-A2L6-01 | Tumor | 8.298 |
| TCGA-WX-AA44-01 | Tumor | 8.298 |
| TCGA-CC-5259-01 | Tumor | 8.262 |
| TCGA-RC-A7SH-01 | Tumor | 8.228 |
| TCGA-G3-A25U-01 | Tumor | 8.227 |
| TCGA-DD-AAEA-01 | Tumor | 8.22 |
| TCGA-EP-A2KB-01 | Tumor | 8.207 |
| TCGA-DD-A4NN-01 | Tumor | 8.189 |
| TCGA-FV-A3I1-01 | Tumor | 8.188 |
| TCGA-DD-AACS-01 | Tumor | 8.166 |
| TCGA-DD-A3A3-01 | Tumor | 8.132 |
| TCGA-GJ-A6C0-01 | Tumor | 8.116 |
| TCGA-DD-AAD8-01 | Tumor | 8.101 |
| TCGA-UB-A7ME-01 | Tumor | 8.099 |
| TCGA-DD-A113-01 | Tumor | 8.097 |
| TCGA-DD-A4ND-01 | Tumor | 8.088 |
| TCGA-DD-AAD2-01 | Tumor | 8.069 |
| TCGA-DD-A39Z-01 | Tumor | 8.067 |
| TCGA-DD-A11C-01 | Tumor | 7.996 |
| TCGA-DD-AAD0-01 | Tumor | 7.996 |
| TCGA-ED-A5KG-01 | Tumor | 7.984 |
| TCGA-DD-A1EF-01 | Tumor | 7.974 |
| TCGA-DD-A73G-01 | Tumor | 7.974 |
| TCGA-DD-A115-01 | Tumor | 7.926 |
| TCGA-DD-AACF-01 | Tumor | 7.925 |
| TCGA-EP-A3JL-01 | Tumor | 7.912 |
| TCGA-KR-A7K8-01 | Tumor | 7.905 |
| TCGA-DD-A73F-01 | Tumor | 7.896 |
| TCGA-G3-AAV3-01 | Tumor | 7.892 |
| TCGA-DD-A1EG-01 | Tumor | 7.866 |
| TCGA-WJ-A86L-01 | Tumor | 7.85 |
| TCGA-ZP-A9D2-01 | Tumor | 7.84 |
| TCGA-CC-A7IF-01 | Tumor | 7.829 |
| TCGA-2Y-A9HA-01 | Tumor | 7.815 |
| TCGA-UB-A7MC-01 | Tumor | 7.799 |
| TCGA-DD-A1EI-01 | Tumor | 7.799 |
| TCGA-4R-AA8I-01 | Tumor | 7.795 |
| TCGA-RC-A7SF-01 | Tumor | 7.793 |
| TCGA-ED-A66X-01 | Tumor | 7.791 |
| TCGA-FV-A23B-01 | Tumor | 7.768 |
| TCGA-MI-A75C-01 | Tumor | 7.76 |
| TCGA-DD-A73B-01 | Tumor | 7.692 |
| TCGA-PD-A5DF-01 | Tumor | 7.682 |
| TCGA-DD-A1EC-01 | Tumor | 7.674 |
| TCGA-RC-A6M4-01 | Tumor | 7.668 |
| TCGA-BW-A5NO-01 | Tumor | 7.666 |
| TCGA-BC-A10Z-01 | Tumor | 7.621 |
| TCGA-2Y-A9H9-01 | Tumor | 7.613 |
| TCGA-DD-AACW-01 | Tumor | 7.611 |
| TCGA-DD-AAEG-01 | Tumor | 7.604 |
| TCGA-DD-A3A5-01 | Tumor | 7.587 |
| TCGA-DD-A11A-01 | Tumor | 7.574 |
| TCGA-DD-A4NA-01 | Tumor | 7.543 |
| TCGA-WX-AA47-01 | Tumor | 7.542 |
| TCGA-FV-A3R2-01 | Tumor | 7.526 |
| TCGA-CC-5261-01 | Tumor | 7.507 |
| TCGA-DD-A3A1-01 | Tumor | 7.495 |
| TCGA-DD-AAVU-01 | Tumor | 7.488 |
| TCGA-UB-A7MD-01 | Tumor | 7.455 |
| TCGA-G3-AAV5-01 | Tumor | 7.434 |
| TCGA-GJ-A3OU-01 | Tumor | 7.428 |
| TCGA-ED-A97K-01 | Tumor | 7.428 |
| TCGA-G3-A3CH-01 | Tumor | 7.427 |
| TCGA-DD-A39X-01 | Tumor | 7.401 |
| TCGA-G3-AAV4-01 | Tumor | 7.38 |
| TCGA-DD-A4NH-01 | Tumor | 7.369 |
| TCGA-G3-A5SM-01 | Tumor | 7.363 |
| TCGA-DD-AACQ-01 | Tumor | 7.36 |
| TCGA-CC-A7IL-01 | Tumor | 7.355 |
| TCGA-BC-A10U-01 | Tumor | 7.35 |
| TCGA-DD-AADL-01 | Tumor | 7.327 |
| TCGA-ED-A8O5-01 | Tumor | 7.311 |
| TCGA-DD-AAE2-01 | Tumor | 7.304 |
| TCGA-2Y-A9GU-01 | Tumor | 7.294 |
| TCGA-DD-AACK-01 | Tumor | 7.291 |
| TCGA-BC-A10T-01 | Tumor | 7.284 |
| TCGA-BW-A5NP-01 | Tumor | 7.251 |
| TCGA-DD-AAVV-01 | Tumor | 7.25 |
| TCGA-RC-A7SB-01 | Tumor | 7.231 |
| TCGA-BD-A3EP-01 | Tumor | 7.226 |
| TCGA-BC-A3KF-01 | Tumor | 7.216 |
| TCGA-DD-A1EE-01 | Tumor | 7.196 |
| TCGA-O8-A75V-01 | Tumor | 7.183 |
| TCGA-MI-A75E-01 | Tumor | 7.179 |
| TCGA-CC-A9FW-01 | Tumor | 7.168 |
| TCGA-KR-A7K2-01 | Tumor | 7.16 |
| TCGA-2Y-A9H4-01 | Tumor | 7.151 |
| TCGA-BC-A5W4-01 | Tumor | 7.149 |
| TCGA-DD-AADK-01 | Tumor | 7.136 |
| TCGA-ED-A7PY-01 | Tumor | 7.13 |
| TCGA-DD-AAEH-01 | Tumor | 7.109 |
| TCGA-DD-A39V-01 | Tumor | 7.084 |
| TCGA-DD-A1EA-01 | Tumor | 7.079 |
| TCGA-GJ-A9DB-01 | Tumor | 7.077 |
| TCGA-DD-A1EH-01 | Tumor | 7.052 |
| TCGA-DD-AACA-01 | Tumor | 7.05 |
| TCGA-ED-A7XP-01 | Tumor | 7.03 |
| TCGA-WQ-AB4B-01 | Tumor | 7.03 |
| TCGA-2Y-A9H5-01 | Tumor | 7.018 |
| TCGA-G3-AAV1-01 | Tumor | 7.016 |
| TCGA-DD-AAED-01 | Tumor | 7.008 |
| TCGA-DD-AAEE-01 | Tumor | 6.974 |
| TCGA-2Y-A9GW-01 | Tumor | 6.958 |
| TCGA-DD-AAVR-01 | Tumor | 6.944 |
| TCGA-DD-A73A-01 | Tumor | 6.942 |
| TCGA-2Y-A9GX-01 | Tumor | 6.933 |
| TCGA-2Y-A9H7-01 | Tumor | 6.926 |
| TCGA-DD-AACD-01 | Tumor | 6.922 |
| TCGA-DD-AAVZ-01 | Tumor | 6.89 |
| TCGA-XR-A8TF-01 | Tumor | 6.861 |
| TCGA-DD-AAE9-01 | Tumor | 6.837 |
| TCGA-G3-A25Z-01 | Tumor | 6.803 |
| TCGA-FV-A2QR-01 | Tumor | 6.797 |
| TCGA-DD-AACI-01 | Tumor | 6.792 |
| TCGA-DD-AAEK-01 | Tumor | 6.785 |
| TCGA-MR-A8JO-01 | Tumor | 6.775 |
| TCGA-K7-A6G5-01 | Tumor | 6.734 |
| TCGA-ES-A2HS-01 | Tumor | 6.715 |
| TCGA-DD-A118-01 | Tumor | 6.712 |
| TCGA-KR-A7K0-01 | Tumor | 6.709 |
| TCGA-BC-A10R-01 | Tumor | 6.689 |
| TCGA-RC-A7SK-01 | Tumor | 6.673 |
| TCGA-DD-AACU-01 | Tumor | 6.67 |
| TCGA-DD-AAVS-01 | Tumor | 6.657 |
| TCGA-DD-AAVP-01 | Tumor | 6.649 |
| TCGA-DD-A1EK-01 | Tumor | 6.628 |
| TCGA-DD-A119-01 | Tumor | 6.602 |
| TCGA-HP-A5MZ-01 | Tumor | 6.6 |
| TCGA-DD-AADY-01 | Tumor | 6.573 |
| TCGA-ZP-A9CV-01 | Tumor | 6.57 |
| TCGA-FV-A3I0-01 | Tumor | 6.56 |
| TCGA-DD-AADP-01 | Tumor | 6.558 |
| TCGA-FV-A496-01 | Tumor | 6.549 |
| TCGA-CC-A7IH-01 | Tumor | 6.514 |
| TCGA-EP-A12J-01 | Tumor | 6.511 |
| TCGA-LG-A6GG-01 | Tumor | 6.509 |
| TCGA-2Y-A9H3-01 | Tumor | 6.497 |
| TCGA-DD-A116-01 | Tumor | 6.486 |
| TCGA-EP-A2KC-01 | Tumor | 6.446 |
| TCGA-MI-A75H-01 | Tumor | 6.441 |
| TCGA-DD-AADJ-01 | Tumor | 6.431 |
| TCGA-2Y-A9HB-01 | Tumor | 6.423 |
| TCGA-5C-A9VH-01 | Tumor | 6.413 |
| TCGA-2Y-A9GZ-01 | Tumor | 6.409 |
| TCGA-XR-A8TC-01 | Tumor | 6.375 |
| TCGA-DD-A3A6-01 | Tumor | 6.373 |
| TCGA-DD-AAVW-01 | Tumor | 6.369 |
| TCGA-DD-AACT-01 | Tumor | 6.369 |
| TCGA-BC-A216-11 | Normal | 6.348 |
| TCGA-DD-AAW2-01 | Tumor | 6.347 |
| TCGA-DD-AADQ-01 | Tumor | 6.327 |
| TCGA-FV-A2QQ-01 | Tumor | 6.286 |
| TCGA-DD-AAW0-01 | Tumor | 6.28 |
| TCGA-ZP-A9D4-01 | Tumor | 6.274 |
| TCGA-DD-AAD3-01 | Tumor | 6.262 |
| TCGA-G3-A3CK-01 | Tumor | 6.259 |
| TCGA-DD-AACE-01 | Tumor | 6.239 |
| TCGA-CC-A9FS-01 | Tumor | 6.2 |
| TCGA-DD-AADI-01 | Tumor | 6.187 |
| TCGA-DD-A3A9-01 | Tumor | 6.168 |
| TCGA-2Y-A9GT-01 | Tumor | 6.13 |
| TCGA-NI-A8LF-01 | Tumor | 6.096 |
| TCGA-DD-AAD1-01 | Tumor | 6.091 |
| TCGA-G3-A3CG-01 | Tumor | 6.082 |
| TCGA-5R-AA1C-01 | Tumor | 6.058 |
| TCGA-DD-AAE1-01 | Tumor | 6.001 |
| TCGA-G3-A5SL-01 | Tumor | 5.997 |
| TCGA-ED-A4XI-01 | Tumor | 5.958 |
| TCGA-ZS-A9CE-01 | Tumor | 5.935 |
| TCGA-BD-A3ER-01 | Tumor | 5.931 |
| TCGA-G3-A25V-01 | Tumor | 5.916 |
| TCGA-DD-AACJ-01 | Tumor | 5.913 |
| TCGA-EP-A26S-01 | Tumor | 5.797 |
| TCGA-DD-AADA-01 | Tumor | 5.794 |
| TCGA-DD-AADG-01 | Tumor | 5.735 |
| TCGA-ZP-A9CY-01 | Tumor | 5.722 |
| TCGA-DD-AAW3-01 | Tumor | 5.679 |
| TCGA-MI-A75G-01 | Tumor | 5.67 |
| TCGA-2Y-A9H1-01 | Tumor | 5.665 |
| TCGA-ES-A2HT-01 | Tumor | 5.665 |
| TCGA-G3-AAV0-01 | Tumor | 5.648 |
| TCGA-DD-AAC9-01 | Tumor | 5.629 |
| TCGA-DD-A4NS-01 | Tumor | 5.584 |
| TCGA-DD-A4NB-01 | Tumor | 5.547 |
| TCGA-ED-A627-01 | Tumor | 5.522 |
| TCGA-DD-A114-11 | Normal | 5.476 |
| TCGA-DD-AAC8-01 | Tumor | 5.433 |
| TCGA-5R-AAAM-01 | Tumor | 5.396 |
| TCGA-G3-A3CI-01 | Tumor | 5.394 |
| TCGA-ES-A2HT-11 | Normal | 5.363 |
| TCGA-DD-A39W-01 | Tumor | 5.356 |
| TCGA-2Y-A9H6-01 | Tumor | 5.353 |
| TCGA-DD-AAEB-01 | Tumor | 5.316 |
| TCGA-ED-A82E-01 | Tumor | 5.315 |
| TCGA-DD-A73E-01 | Tumor | 5.302 |
| TCGA-G3-A6UC-01 | Tumor | 5.279 |
| TCGA-3K-AAZ8-01 | Tumor | 5.256 |
| TCGA-DD-AACN-01 | Tumor | 5.24 |
| TCGA-DD-A4NG-01 | Tumor | 5.216 |
| TCGA-G3-A7M7-01 | Tumor | 5.176 |
| TCGA-DD-AACY-01 | Tumor | 5.166 |
| TCGA-DD-A1EB-01 | Tumor | 5.165 |
| TCGA-ZS-A9CD-01 | Tumor | 5.152 |
| TCGA-DD-A3A8-01 | Tumor | 5.142 |
| TCGA-G3-A7M5-01 | Tumor | 5.116 |
| TCGA-DD-A73D-01 | Tumor | 5.058 |
| TCGA-DD-A3A4-01 | Tumor | 5.056 |
| TCGA-DD-AAE7-01 | Tumor | 5.05 |
| TCGA-BC-A69I-01 | Tumor | 5.038 |
| TCGA-DD-A11B-01 | Tumor | 4.984 |
| TCGA-DD-AADS-01 | Tumor | 4.955 |
| TCGA-LG-A9QC-01 | Tumor | 4.953 |
| TCGA-DD-A39Z-11 | Normal | 4.886 |
| TCGA-DD-A11D-01 | Tumor | 4.861 |
| TCGA-DD-A4NK-01 | Tumor | 4.855 |
| TCGA-5R-AA1D-01 | Tumor | 4.84 |
| TCGA-BC-A10Q-11 | Normal | 4.836 |
| TCGA-DD-AADU-01 | Tumor | 4.829 |
| TCGA-DD-A73C-01 | Tumor | 4.809 |
| TCGA-FV-A3R3-01 | Tumor | 4.803 |
| TCGA-BC-A10X-01 | Tumor | 4.798 |
| TCGA-DD-A1EI-11 | Normal | 4.786 |
| TCGA-ZP-A9D1-01 | Tumor | 4.74 |
| TCGA-NI-A4U2-01 | Tumor | 4.737 |
| TCGA-DD-A4NO-01 | Tumor | 4.731 |
| TCGA-BD-A2L6-11 | Normal | 4.703 |
| TCGA-ZP-A9D0-01 | Tumor | 4.67 |
| TCGA-DD-A4NV-01 | Tumor | 4.653 |
| TCGA-DD-AAVX-01 | Tumor | 4.649 |
| TCGA-G3-A3CJ-01 | Tumor | 4.624 |
| TCGA-CC-A9FV-01 | Tumor | 4.604 |
| TCGA-DD-A4NI-01 | Tumor | 4.569 |
| TCGA-XR-A8TE-01 | Tumor | 4.531 |
| TCGA-DD-AAW1-01 | Tumor | 4.377 |
| TCGA-MR-A520-01 | Tumor | 4.322 |
| TCGA-LG-A9QD-01 | Tumor | 4.313 |
| TCGA-BC-A110-01 | Tumor | 4.298 |
| TCGA-DD-A116-11 | Normal | 4.297 |
| TCGA-T1-A6J8-01 | Tumor | 4.28 |
| TCGA-BC-A10S-01 | Tumor | 4.264 |
| TCGA-HP-A5N0-01 | Tumor | 4.17 |
| TCGA-DD-A4NL-01 | Tumor | 4.027 |
| TCGA-G3-AAV2-01 | Tumor | 4.026 |
| TCGA-FV-A23B-11 | Normal | 3.987 |
| TCGA-FV-A2QR-11 | Normal | 3.945 |
| TCGA-EP-A3RK-11 | Normal | 3.922 |
| TCGA-RC-A6M5-01 | Tumor | 3.917 |
| TCGA-DD-AAE3-01 | Tumor | 3.858 |
| TCGA-K7-A5RF-01 | Tumor | 3.729 |
| TCGA-DD-AAVY-01 | Tumor | 3.727 |
| TCGA-DD-A11C-11 | Normal | 3.688 |
| TCGA-DD-A118-11 | Normal | 3.68 |
| TCGA-DD-A1EE-11 | Normal | 3.664 |
| TCGA-DD-A3A3-11 | Normal | 3.651 |
| TCGA-G3-A7M8-01 | Tumor | 3.636 |
| TCGA-EP-A12J-11 | Normal | 3.609 |
| TCGA-DD-A3A1-11 | Normal | 3.602 |
| TCGA-DD-A1EH-11 | Normal | 3.54 |
| TCGA-DD-A1EG-11 | Normal | 3.498 |
| TCGA-BC-A10T-11 | Normal | 3.483 |
| TCGA-DD-A1EJ-11 | Normal | 3.48 |
| TCGA-2Y-A9GV-01 | Tumor | 3.479 |
| TCGA-DD-A3A2-01 | Tumor | 3.366 |
| TCGA-BC-A10X-11 | Normal | 3.362 |
| TCGA-DD-A11A-11 | Normal | 3.344 |
| TCGA-DD-A4NP-01 | Tumor | 3.324 |
| TCGA-DD-A39X-11 | Normal | 3.31 |
| TCGA-FV-A3R2-11 | Normal | 3.309 |
| TCGA-DD-A1EL-11 | Normal | 3.308 |
| TCGA-G3-A5SK-01 | Tumor | 3.236 |
| TCGA-ZS-A9CG-01 | Tumor | 3.206 |
| TCGA-DD-A39V-11 | Normal | 3.189 |
| TCGA-DD-A11B-11 | Normal | 3.161 |
| TCGA-BC-A10Y-11 | Normal | 3.109 |
| TCGA-G3-A3CH-11 | Normal | 3.016 |
| TCGA-DD-A4NF-01 | Tumor | 2.999 |
| TCGA-UB-AA0V-01 | Tumor | 2.989 |
| TCGA-DD-A3A5-11 | Normal | 2.984 |
| TCGA-DD-A119-11 | Normal | 2.928 |
| TCGA-WX-AA46-01 | Tumor | 2.927 |
| TCGA-DD-A1EC-11 | Normal | 2.92 |
| TCGA-DD-A113-11 | Normal | 2.834 |
| TCGA-BC-A10W-11 | Normal | 2.81 |
| TCGA-DD-A39W-11 | Normal | 2.798 |
| TCGA-DD-A3A4-11 | Normal | 2.793 |
| TCGA-DD-A1ED-01 | Tumor | 2.793 |
| TCGA-DD-A3A2-11 | Normal | 2.72 |
| TCGA-DD-A11D-11 | Normal | 2.563 |
| TCGA-DD-A3A6-11 | Normal | 2.402 |
| TCGA-DD-A3A8-11 | Normal | 2.392 |
| TCGA-FV-A3I0-11 | Normal | 2.203 |
| TCGA-BD-A3EP-11 | Normal | 2.203 |
| TCGA-DD-A1EB-11 | Normal | 2.105 |
| TCGA-BC-A10R-11 | Normal | 1.91 |
| TCGA-EP-A26S-11 | Normal | 1.714 |
| TCGA-BC-A10Z-11 | Normal | 1.696 |
| TCGA-FV-A3I1-11 | Normal | 1.512 |
| TCGA-BC-A10U-11 | Normal | 1.393 |

**Table S13 Co-expression data of miR-4484 and KIF2C in TCGA-LIHC database**

| sample_ID | Tissue type | miR_4484 | KIF2C |
| --- | --- | --- | --- |
| TCGA-2Y-A9GS-01 | Tumor | 0.406726042 | 9.102 |
| TCGA-2Y-A9GT-01 | Tumor | 0.383496944 | 6.13 |
| TCGA-2Y-A9GU-01 | Tumor | 0.356854839 | 7.294 |
| TCGA-2Y-A9GW-01 | Tumor | 1.009470438 | 6.958 |
| TCGA-2Y-A9GX-01 | Tumor | 0.856203296 | 6.933 |
| TCGA-2Y-A9H0-01 | Tumor | 0.827419079 | 9.53 |
| TCGA-2Y-A9H1-01 | Tumor | 0.346925128 | 5.665 |
| TCGA-2Y-A9H4-01 | Tumor | 0.30183461 | 7.151 |
| TCGA-2Y-A9H5-01 | Tumor | 0.45123802 | 7.018 |
| TCGA-2Y-A9H7-01 | Tumor | 0.857996914 | 6.926 |
| TCGA-2Y-A9H9-01 | Tumor | 1.075575757 | 7.613 |
| TCGA-2Y-A9HB-01 | Tumor | 0.295650153 | 6.423 |
| TCGA-3K-AAZ8-01 | Tumor | 0.676003763 | 5.256 |
| TCGA-4R-AA8I-01 | Tumor | 1.088742708 | 7.795 |
| TCGA-5C-A9VG-01 | Tumor | 0.537443187 | 8.786 |
| TCGA-5R-AA1D-01 | Tumor | 1.643389835 | 4.84 |
| TCGA-5R-AAAM-01 | Tumor | 0.275681737 | 5.396 |
| TCGA-BC-4072-01 | Tumor | 0.615699723 | 9.111 |
| TCGA-BC-4073-01 | Tumor | 0.33402931 | 9.295 |
| TCGA-BC-A10Q-01 | Tumor | 0.998537087 | 8.299 |
| TCGA-BC-A10S-01 | Tumor | 0.390406854 | 4.264 |
| TCGA-BC-A10U-01 | Tumor | 0.365461867 | 7.35 |
| TCGA-BC-A10X-01 | Tumor | 0.464009495 | 4.798 |
| TCGA-BC-A10Y-01 | Tumor | 0.805868593 | 8.973 |
| TCGA-BC-A10Z-01 | Tumor | 0.762875192 | 7.621 |
| TCGA-BC-A110-01 | Tumor | 0.421997198 | 4.298 |
| TCGA-BC-A216-01 | Tumor | 1.926140264 | 10.66 |
| TCGA-BC-A217-01 | Tumor | 0.192543905 | 9.104 |
| TCGA-BC-A3KF-01 | Tumor | 0.734633758 | 7.216 |
| TCGA-BC-A3KG-01 | Tumor | 2.632354775 | 9.878 |
| TCGA-BC-A5W4-01 | Tumor | 0.320711101 | 7.149 |
| TCGA-BC-A8YO-01 | Tumor | 0.311247337 | 9.576 |
| TCGA-BD-A2L6-01 | Tumor | 1.069913847 | 8.298 |
| TCGA-BD-A3EP-01 | Tumor | 0.297001197 | 7.226 |
| TCGA-BW-A5NO-01 | Tumor | 0.581257716 | 7.666 |
| TCGA-BW-A5NQ-01 | Tumor | 1.433368647 | 10.39 |
| TCGA-CC-5260-01 | Tumor | 0.444687207 | 8.566 |
| TCGA-CC-5261-01 | Tumor | 0.394261689 | 7.507 |
| TCGA-CC-5262-01 | Tumor | 0.529312229 | 8.737 |
| TCGA-CC-5264-01 | Tumor | 0.682339571 | 10.17 |
| TCGA-CC-A123-01 | Tumor | 0.558756451 | 8.852 |
| TCGA-CC-A3M9-01 | Tumor | 0.275659094 | 11.1 |
| TCGA-CC-A3MA-01 | Tumor | 0.389887253 | 9.661 |
| TCGA-CC-A3MB-01 | Tumor | 1.536766609 | 8.995 |
| TCGA-CC-A3MC-01 | Tumor | 0.345375936 | 8.588 |
| TCGA-CC-A7IE-01 | Tumor | 0.469001338 | 9.117 |
| TCGA-CC-A7IF-01 | Tumor | 0.98458133 | 7.829 |
| TCGA-CC-A7IL-01 | Tumor | 0.64138048 | 7.355 |
| TCGA-CC-A8HT-01 | Tumor | 0.747022114 | 10.44 |
| TCGA-CC-A9FS-01 | Tumor | 1.180114754 | 6.2 |
| TCGA-CC-A9FU-01 | Tumor | 0.39443402 | 9.695 |
| TCGA-CC-A9FV-01 | Tumor | 0.357238941 | 4.604 |
| TCGA-CC-A9FW-01 | Tumor | 0.872950866 | 7.168 |
| TCGA-DD-A113-01 | Tumor | 0.376958225 | 8.097 |
| TCGA-DD-A114-01 | Tumor | 0.313395629 | 9.805 |
| TCGA-DD-A115-01 | Tumor | 0.698886098 | 7.926 |
| TCGA-DD-A116-01 | Tumor | 0.650221379 | 6.486 |
| TCGA-DD-A118-01 | Tumor | 0.602599404 | 6.712 |
| TCGA-DD-A11B-01 | Tumor | 0.363799053 | 4.984 |
| TCGA-DD-A11C-01 | Tumor | 1.13659762 | 7.996 |
| TCGA-DD-A11D-01 | Tumor | 1.121625515 | 4.861 |
| TCGA-DD-A1EA-01 | Tumor | 1.226462289 | 7.079 |
| TCGA-DD-A1EB-01 | Tumor | 1.216302749 | 5.165 |
| TCGA-DD-A1EC-01 | Tumor | 2.890241023 | 7.674 |
| TCGA-DD-A1ED-01 | Tumor | 1.098346788 | 2.793 |
| TCGA-DD-A1EE-01 | Tumor | 0.299269279 | 7.196 |
| TCGA-DD-A1EF-01 | Tumor | 0.422930494 | 7.974 |
| TCGA-DD-A1EG-01 | Tumor | 0.204054303 | 7.866 |
| TCGA-DD-A1EH-01 | Tumor | 1.136378439 | 7.052 |
| TCGA-DD-A1EK-01 | Tumor | 0.342858499 | 6.628 |
| TCGA-DD-A1EL-01 | Tumor | 0.548138684 | 9.121 |
| TCGA-DD-A39V-01 | Tumor | 0.189165433 | 7.084 |
| TCGA-DD-A39W-01 | Tumor | 0.918241194 | 5.356 |
| TCGA-DD-A39X-01 | Tumor | 0.389346536 | 7.401 |
| TCGA-DD-A39Y-01 | Tumor | 0.300251438 | 10.13 |
| TCGA-DD-A39Z-01 | Tumor | 0.469578655 | 8.067 |
| TCGA-DD-A3A4-01 | Tumor | 1.906234886 | 5.056 |
| TCGA-DD-A3A5-01 | Tumor | 0.214538899 | 7.587 |
| TCGA-DD-A3A6-01 | Tumor | 0.746102905 | 6.373 |
| TCGA-DD-A3A7-01 | Tumor | 0.296873196 | 9.028 |
| TCGA-DD-A3A8-01 | Tumor | 0.85363882 | 5.142 |
| TCGA-DD-A4NB-01 | Tumor | 1.265895917 | 5.547 |
| TCGA-DD-A4ND-01 | Tumor | 0.451361474 | 8.088 |
| TCGA-DD-A4NI-01 | Tumor | 0.647156069 | 4.569 |
| TCGA-DD-A4NP-01 | Tumor | 0.367245803 | 3.324 |
| TCGA-DD-A4NV-01 | Tumor | 0.871055864 | 4.653 |
| TCGA-DD-A73A-01 | Tumor | 1.09406645 | 6.942 |
| TCGA-DD-A73D-01 | Tumor | 0.183024016 | 5.058 |
| TCGA-DD-AAC9-01 | Tumor | 0.214504085 | 5.629 |
| TCGA-DD-AACA-01 | Tumor | 0.297183198 | 7.05 |
| TCGA-DD-AACA-02 | Tumor | 0.677778828 | 7.979 |
| TCGA-DD-AACB-01 | Tumor | 0.280621427 | 8.848 |
| TCGA-DD-AACE-01 | Tumor | 0.805684551 | 6.239 |
| TCGA-DD-AACF-01 | Tumor | 0.550525417 | 7.925 |
| TCGA-DD-AACH-01 | Tumor | 0.144896069 | 9.668 |
| TCGA-DD-AACI-01 | Tumor | 0.21547732 | 6.792 |
| TCGA-DD-AACJ-01 | Tumor | 0.236943189 | 5.913 |
| TCGA-DD-AACN-01 | Tumor | 0.586543789 | 5.24 |
| TCGA-DD-AACO-01 | Tumor | 0.333948047 | 8.616 |
| TCGA-DD-AACP-01 | Tumor | 0.268635602 | 9.411 |
| TCGA-DD-AACS-01 | Tumor | 0.227068236 | 8.166 |
| TCGA-DD-AACU-01 | Tumor | 0.255536989 | 6.67 |
| TCGA-DD-AACW-01 | Tumor | 0.33589938 | 7.611 |
| TCGA-DD-AACX-01 | Tumor | 0.837286919 | 8.547 |
| TCGA-DD-AACY-01 | Tumor | 0.288730632 | 5.166 |
| TCGA-DD-AACZ-01 | Tumor | 0.389561305 | 9.296 |
| TCGA-DD-AAD1-01 | Tumor | 0.452339237 | 6.091 |
| TCGA-DD-AAD2-01 | Tumor | 0.302617357 | 8.069 |
| TCGA-DD-AAD3-01 | Tumor | 0.162623833 | 6.262 |
| TCGA-DD-AADA-01 | Tumor | 0.231464359 | 5.794 |
| TCGA-DD-AADC-01 | Tumor | 0.260236473 | 9.61 |
| TCGA-DD-AADF-01 | Tumor | 0.654353963 | 8.599 |
| TCGA-DD-AADL-01 | Tumor | 0.394530605 | 7.327 |
| TCGA-DD-AADM-01 | Tumor | 0.247695449 | 8.309 |
| TCGA-DD-AADP-01 | Tumor | 0.251371289 | 6.558 |
| TCGA-DD-AADQ-01 | Tumor | 0.608705176 | 6.327 |
| TCGA-DD-AADR-01 | Tumor | 1.619249598 | 8.392 |
| TCGA-DD-AADS-01 | Tumor | 0.206474441 | 4.955 |
| TCGA-DD-AADU-01 | Tumor | 1.312359643 | 4.829 |
| TCGA-DD-AADW-01 | Tumor | 0.38205076 | 8.811 |
| TCGA-DD-AADY-01 | Tumor | 0.778443258 | 6.573 |
| TCGA-DD-AAE1-01 | Tumor | 0.499706616 | 6.001 |
| TCGA-DD-AAE2-01 | Tumor | 0.82398816 | 7.304 |
| TCGA-DD-AAE4-01 | Tumor | 0.553122574 | 8.537 |
| TCGA-DD-AAE6-01 | Tumor | 0.293629452 | 9.026 |
| TCGA-DD-AAEA-01 | Tumor | 0.52954812 | 8.22 |
| TCGA-DD-AAEB-01 | Tumor | 0.226434544 | 5.316 |
| TCGA-DD-AAED-01 | Tumor | 0.292562678 | 7.008 |
| TCGA-DD-AAEE-01 | Tumor | 0.860417787 | 6.974 |
| TCGA-DD-AAEG-01 | Tumor | 0.272674196 | 7.604 |
| TCGA-DD-AAEH-01 | Tumor | 0.228391436 | 7.109 |
| TCGA-DD-AAEI-01 | Tumor | 0.40287495 | 8.401 |
| TCGA-DD-AAEK-01 | Tumor | 0.485499991 | 6.785 |
| TCGA-DD-AAVQ-01 | Tumor | 0.309851383 | 8.346 |
| TCGA-DD-AAVU-01 | Tumor | 0.188678169 | 7.488 |
| TCGA-DD-AAVV-01 | Tumor | 0.157768111 | 7.25 |
| TCGA-DD-AAVZ-01 | Tumor | 0.295199916 | 6.89 |
| TCGA-DD-AAW0-01 | Tumor | 0.414883483 | 6.28 |
| TCGA-ED-A627-01 | Tumor | 0.361722327 | 5.522 |
| TCGA-ED-A7PX-01 | Tumor | 0.946784001 | 8.511 |
| TCGA-ED-A7PY-01 | Tumor | 0.945445897 | 7.13 |
| TCGA-ED-A7PZ-01 | Tumor | 1.075963835 | 8.74 |
| TCGA-ED-A7XO-01 | Tumor | 1.606684789 | 8.828 |
| TCGA-ED-A7XP-01 | Tumor | 0.479193303 | 7.03 |
| TCGA-ED-A82E-01 | Tumor | 0.623653391 | 5.315 |
| TCGA-ED-A8O5-01 | Tumor | 0.394734727 | 7.311 |
| TCGA-ED-A8O6-01 | Tumor | 0.422985374 | 9.151 |
| TCGA-ED-A97K-01 | Tumor | 0.318057639 | 7.428 |
| TCGA-EP-A12J-01 | Tumor | 0.254960414 | 6.511 |
| TCGA-EP-A26S-01 | Tumor | 0.838660577 | 5.797 |
| TCGA-EP-A3JL-01 | Tumor | 0.225985612 | 7.912 |
| TCGA-EP-A3RK-01 | Tumor | 0.422216851 | 8.73 |
| TCGA-ES-A2HS-01 | Tumor | 0.480632213 | 6.715 |
| TCGA-FV-A23B-01 | Tumor | 0.662187266 | 7.768 |
| TCGA-FV-A2QR-01 | Tumor | 0.560817651 | 6.797 |
| TCGA-FV-A3I1-01 | Tumor | 0.506493282 | 8.188 |
| TCGA-FV-A4ZQ-01 | Tumor | 0.488050146 | 9.106 |
| TCGA-G3-A25S-01 | Tumor | 0.532211216 | 8.792 |
| TCGA-G3-A25T-01 | Tumor | 0.457080472 | 9.039 |
| TCGA-G3-A25U-01 | Tumor | 1.874678556 | 8.227 |
| TCGA-G3-A25V-01 | Tumor | 0.207389382 | 5.916 |
| TCGA-G3-A25Y-01 | Tumor | 0.739202633 | 8.528 |
| TCGA-G3-A25Z-01 | Tumor | 0.248212987 | 6.803 |
| TCGA-G3-A3CG-01 | Tumor | 1.891954946 | 6.082 |
| TCGA-G3-A3CH-01 | Tumor | 1.160238037 | 7.427 |
| TCGA-G3-A3CI-01 | Tumor | 1.353698174 | 5.394 |
| TCGA-G3-A3CJ-01 | Tumor | 0.373778486 | 4.624 |
| TCGA-G3-A3CK-01 | Tumor | 0.29363416 | 6.259 |
| TCGA-G3-A5SI-01 | Tumor | 0.609439161 | 9.374 |
| TCGA-G3-A5SJ-01 | Tumor | 0.419019967 | 8.97 |
| TCGA-G3-A5SK-01 | Tumor | 0.412562598 | 3.236 |
| TCGA-G3-A6UC-01 | Tumor | 0.701789449 | 5.279 |
| TCGA-G3-A7M7-01 | Tumor | 0.884453351 | 5.176 |
| TCGA-G3-A7M9-01 | Tumor | 0.528873328 | 11.09 |
| TCGA-G3-AAUZ-01 | Tumor | 0.536685557 | 8.332 |
| TCGA-G3-AAV1-01 | Tumor | 0.380268422 | 7.016 |
| TCGA-G3-AAV2-01 | Tumor | 0.781091085 | 4.026 |
| TCGA-G3-AAV4-01 | Tumor | 0.512143939 | 7.38 |
| TCGA-G3-AAV5-01 | Tumor | 0.698509211 | 7.434 |
| TCGA-HP-A5MZ-01 | Tumor | 0.307303778 | 6.6 |
| TCGA-HP-A5N0-01 | Tumor | 0.635574246 | 4.17 |
| TCGA-K7-A5RG-01 | Tumor | 0.376647123 | 8.904 |
| TCGA-K7-AAU7-01 | Tumor | 0.863186785 | 8.714 |
| TCGA-KR-A7K7-01 | Tumor | 1.2442039 | 9.339 |
| TCGA-KR-A7K8-01 | Tumor | 1.214188233 | 7.905 |
| TCGA-LG-A6GG-01 | Tumor | 0.539595587 | 6.509 |
| TCGA-LG-A9QC-01 | Tumor | 0.325584441 | 4.953 |
| TCGA-MI-A75C-01 | Tumor | 0.424451322 | 7.76 |
| TCGA-MI-A75E-01 | Tumor | 1.085160841 | 7.179 |
| TCGA-MI-A75G-01 | Tumor | 1.686776154 | 5.67 |
| TCGA-MI-A75I-01 | Tumor | 0.563073177 | 8.476 |
| TCGA-MR-A520-01 | Tumor | 1.102139407 | 4.322 |
| TCGA-MR-A8JO-01 | Tumor | 0.700325186 | 6.775 |
| TCGA-NI-A4U2-01 | Tumor | 1.024479261 | 4.737 |
| TCGA-NI-A8LF-01 | Tumor | 1.04521928 | 6.096 |
| TCGA-O8-A75V-01 | Tumor | 0.368008395 | 7.183 |
| TCGA-QA-A7B7-01 | Tumor | 0.311682133 | 9.786 |
| TCGA-RC-A6M3-01 | Tumor | 0.614789926 | 9.611 |
| TCGA-RC-A6M5-01 | Tumor | 0.199562421 | 3.917 |
| TCGA-RC-A6M6-01 | Tumor | 0.331236279 | 9.817 |
| TCGA-RC-A7S9-01 | Tumor | 0.974449283 | 9.241 |
| TCGA-RC-A7SB-01 | Tumor | 0.543586946 | 7.231 |
| TCGA-RC-A7SF-01 | Tumor | 0.341816142 | 7.793 |
| TCGA-RC-A7SK-01 | Tumor | 0.475385845 | 6.673 |
| TCGA-T1-A6J8-01 | Tumor | 0.230036965 | 4.28 |
| TCGA-UB-A7MB-01 | Tumor | 0.818649355 | 8.955 |
| TCGA-UB-A7MC-01 | Tumor | 0.60090341 | 7.799 |
| TCGA-UB-A7MD-01 | Tumor | 0.237221053 | 7.455 |
| TCGA-UB-A7ME-01 | Tumor | 0.462407319 | 8.099 |
| TCGA-UB-AA0U-01 | Tumor | 0.215049823 | 8.671 |
| TCGA-UB-AA0V-01 | Tumor | 0.532030636 | 2.989 |
| TCGA-WQ-A9G7-01 | Tumor | 1.184107605 | 9.225 |
| TCGA-WQ-AB4B-01 | Tumor | 0.270002599 | 7.03 |
| TCGA-WX-AA46-01 | Tumor | 1.354539611 | 2.927 |
| TCGA-WX-AA47-01 | Tumor | 1.534825092 | 7.542 |
| TCGA-XR-A8TC-01 | Tumor | 0.616461213 | 6.375 |
| TCGA-XR-A8TF-01 | Tumor | 0.684781941 | 6.861 |
| TCGA-YA-A8S7-01 | Tumor | 0.235055484 | 9.579 |
| TCGA-ZP-A9CV-01 | Tumor | 0.25067058 | 6.57 |
| TCGA-ZP-A9CY-01 | Tumor | 0.84250905 | 5.722 |
| TCGA-ZP-A9CZ-01 | Tumor | 0.379501195 | 9.165 |
| TCGA-ZP-A9D1-01 | Tumor | 0.237552705 | 4.74 |
| TCGA-ZP-A9D4-01 | Tumor | 0.748058424 | 6.274 |
| TCGA-ZS-A9CE-01 | Tumor | 0.874662426 | 5.935 |
| TCGA-ZS-A9CF-01 | Tumor | 1.137074586 | 8.913 |
| TCGA-ZS-A9CF-02 | Tumor | 0.249425923 | 8.862 |
| TCGA-ZS-A9CG-01 | Tumor | 0.900551916 | 3.206 |
