# Supplementary material for: miR-4484 suppresses hepatocellular carcinoma progression via targeting KIF2C
Source: RNA Biol. 2025 Oct 2;22(1):1–20. doi: 10.1080/15476286.2025.2569192 (PMC12498537; doi:10.1080/15476286.2025.2569192)
Supplement: Table S1 miR4484 inhibitor mimics sequence.docx [file KRNB_A_2569192_SM1641.docx]

| **>hsa-miR-4484 MIMAT0019018** |
| --- |
| 5’-AAAAGGCGGGAGAAGCCCCA-3’ |
| **hsa-miR-4484 mimic sequence:** |
| 5’-AAAAGGCGGGAGAAGCCCCA-3’ |
| **hsa-miR-4484 Inhibitor sequence:** |
| 5’-UGGGGCUUCUCCCGCCUUUU-3’ |
| **miR-4484 N.C sequence：** |
| 5’-UUCUCCGAACGUGUCACGUTT-3’ |
