# Supplementary material for: miR-4484 suppresses hepatocellular carcinoma progression via targeting KIF2C
Source: RNA Biol. 2025 Oct 2;22(1):1–20. doi: 10.1080/15476286.2025.2569192 (PMC12498537; doi:10.1080/15476286.2025.2569192)
Supplement: Table S4 KIF2C plasmid vetor construct.docx [file KRNB_A_2569192_SM1640.docx]

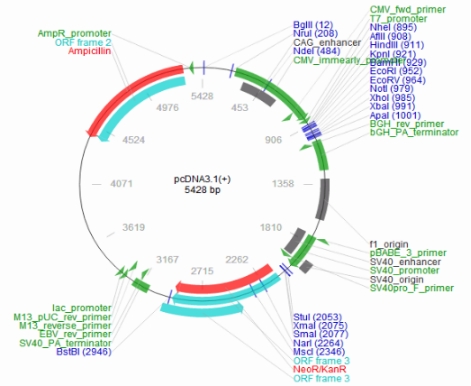


map of pCDNA3.1(+) vector

KIF2C-F：5’-CCCAAGCTTATGGCCATGGACTCGTCG-3’ (Hind III)

KIF2C-R：5’-CGGAATTCTCACTGGGGCCGTTTCTTG-3’ (EcoR I)
