# Supplementary material for: miR-4484 suppresses hepatocellular carcinoma progression via targeting KIF2C
Source: RNA Biol. 2025 Oct 2;22(1):1–20. doi: 10.1080/15476286.2025.2569192 (PMC12498537; doi:10.1080/15476286.2025.2569192)
Supplement: Table S2 primers for qPCR.docx [file KRNB_A_2569192_SM1639.docx]

1. The related primers in qPCR were as followed.
2. reverse-transcription primer:

5'-GTCGTATCCAGTGCAGGGTCCGAGGTATTCGCACTGGATACGACTGGGGC-3';

1. miR-4484

Forward: 5'-GCGAAAAGGCGGGAGAA-3';

Reverse: 5'-AGTGCAGGGTCCGAGGTATT-3';

1. U6 small nuclear 1 (RNU6-1)

Forward: 5'-CTCGCTTCGGCAGCACA-3'

Reverse: 5'-AACGCTTCACGAATTTGCGT-3';

1. KIF2C

Forward: 5'-CCCAAGCTTATGGCCATGGACTCGTCG-3';

Reverse: 5'-CGGAATTCTCACTGGGGCCGTTTCTTG-3';

1. GAPDH

Forward: 5'-AATCCCATCACCATCTTC-3';

Reverse: 5'-AGGCTGTTGTCATACTTC-3'.
