# Supplementary material for: miR-4484 suppresses hepatocellular carcinoma progression via targeting KIF2C
Source: RNA Biol. 2025 Oct 2;22(1):1–20. doi: 10.1080/15476286.2025.2569192 (PMC12498537; doi:10.1080/15476286.2025.2569192)
Supplement: Table S7 miR4484 expr and pathological stages.docx [file KRNB_A_2569192_SM1636.docx]

**Relationship between miR-4484 expression level and pathological stages**

| Pathological type | Total number | Group(n) | | | P-Value |
| --- | --- | --- | --- | --- | --- |
| Histologic_grade  (Mean ± SD) | 226 | G1 (n = 33) | G2 (n = 109) | G3&G4 (n = 84) | 0.0279 |
|  |  | 0.72 ± 0.34 | 0.61 ± 0.40 | 0.61 ± 0.52 |  |
| T stage  (Mean ± SD) | 229 | T1 (n = 115) | T2 (n = 59) | T3&T4 (n = 55) | 0.0259 |
|  |  | 0.58 ± 0.42 | 0.66 ± 0.45 | 0.72 ± 0.44 |  |
| N stage  (Mean ± SD) | 228 | N0 (n = 44) | N1&NX  (n = 184) |  | 0.3047 |
|  |  | 0.55 ± 0.33 | 0.65 ± 0.46 |  |  |
| M stage  (Mean ± SD) | 229 | M0 (n = 167) | M1&Mx  (n = 62) |  | 0.316 |
|  |  | 0.63 ± 0.47 | 0.63 ± 0.34 |  |  |
| Pathologic stage  (Mean ± SD) | 214 | Stage I (n = 107) | Stage II  (n = 54) | Stage III&IV  (n = 53) | 0.1114 |
|  |  | 0.59 ± 0.43 | 0.66 ± 0.46 | 0.7030 ± 0.4458 |  |
|  |  | Stage III &IV VS. Stage I | | | 0.0307 |
