# Supplementary material for: miR-4484 suppresses hepatocellular carcinoma progression via targeting KIF2C
Source: RNA Biol. 2025 Oct 2;22(1):1–20. doi: 10.1080/15476286.2025.2569192 (PMC12498537; doi:10.1080/15476286.2025.2569192)
Supplement: Table S3 KIF2C sequence.docx [file KRNB_A_2569192_SM1632.docx]

**1.The wtKIF2C 3'-UTR is as followed:**

CGACTGCAAATAAAAATCTGTTTGGTTTGACACCCAGCCTCTTCCCTGGCCCTCCCCAGAGAACTTTGGGTACCTGGTGGGTCTAGGCAGGGTCTGAGCTGGGACAGGTTCTGGTAAATGCCAAGTATGGGGGCATCTGGGCCCAGGGCAGCTGGGGAGGGGGTCAGAGTGACATGGGACACTCCTTTTCTGTTCCTCAGTTGTCGCCCTCACGAGAGGAAGGAGCTCTTAGTTACCCTTTTGTGTTGCCCTTCTTTCCATCAAGGGGAATGTTCTCAGCATAGAGCTTTCTCCGCAGCATCCTGCCTGCGTGGACTGGCTGCTAATGGAGAGCTCCCTGGGGTTGTCCTGGCTCTGGGGAGAGAGACGGA*GCCTTT*AGTACAGCTATCTGCTGGCTCTAAACCTTCTA*CGCCTTT*GGGCCGAGCACTGAATGTCTTGTACTTTAAAAAAATGTTTCTGAGACCTCTTTCTACTTTACTGTCTCCCTAGAGATCCTAGAGGATCCCTACTGTTTTCTGTTTTATGTGTTTATACATTGTATGTAACAATAAAGAGAAAAAATAAATCAGCTGTTTAAGTGTGTGGAAAAAAAAAAAAAAAAAA

**2.The mutKIF2C 3'-UTR is as followed:**

CGACTGCAAATAAAAATCTGTTTGGTTTGACACCCAGCCTCTTCCCTGGCCCTCCCCAGAGAACTTTGGGTACCTGGTGGGTCTAGGCAGGGTCTGAGCTGGGACAGGTTCTGGTAAATGCCAAGTATGGGGGCATCTGGGCCCAGGGCAGCTGGGGAGGGGGTCAGAGTGACATGGGACACTCCTTTTCTGTTCCTCAGTTGTCGCCCTCACGAGAGGAAGGAGCTCTTAGTTACCCTTTTGTGTTGCCCTTCTTTCCATCAAGGGGAATGTTCTCAGCATAGAGCTTTCTCCGCAGCATCCTGCCTGCGTGGACTGGCTGCTAATGGAGAGCTCCCTGGGGTTGTCCTGGCTCTGGGGAGAGAGACGGA*AATGGC*AGTACAGCTATCTGCTGGCTCTAAACCTTCTA*TAATGGC*GGGCCGAGCACTGAATGTCTTGTACTTTAAAAAAATGTTTCTGAGACCTCTTTCTACTTTACTGTCTCCCTAGAGATCCTAGAGGATCCCTACTGTTTTCTGTTTTATGTGTTTATACATTGTATGTAACAATAAAGAGAAAAAATAAATCAGCTGTTTAAGTGTGTGGAAAAAAAAAAAAAAAAAA

Note: The italic sequence with underline and yellow background represents the combing region.
